# An essential thioredoxin-type protein of *Trypanosoma brucei* acts as redox-regulated mitochondrial chaperone

**DOI:** 10.1371/journal.ppat.1008065

**Published:** 2019-09-26

**Authors:** Rachel B. Currier, Kathrin Ulrich, Alejandro E. Leroux, Natalie Dirdjaja, Matías Deambrosi, Mariana Bonilla, Yasar Luqman Ahmed, Lorenz Adrian, Haike Antelmann, Ursula Jakob, Marcelo A. Comini, R. Luise Krauth-Siegel

**Affiliations:** 1 Biochemie-Zentrum der Universität Heidelberg (BZH), Heidelberg, Germany; 2 Department of Molecular, Cellular, and Developmental Biology, University of Michigan, Ann Arbor, Michigan, United States of America; 3 Group Redox Biology of Trypanosomes, Institut Pasteur de Montevideo, Montevideo, Uruguay; 4 Laboratorio de Fisicoquímica Biológica, Instituto de Química Biológica, Facultad de Ciencias, Universidad de la República, Montevideo, Uruguay; 5 Department of Isotope Biogeochemistry, Helmholtz Centre for Environmental Research–UFZ, Leipzig, Germany; 6 Fachgebiet Geobiotechnologie, Technische Universität Berlin, Berlin, Germany; 7 Institut für Biologie-Mikrobiologie, Freie Universität Berlin, Berlin, Germany; University of Glasgow, UNITED KINGDOM

## Abstract

Most known thioredoxin-type proteins (Trx) participate in redox pathways, using two highly conserved cysteine residues to catalyze thiol-disulfide exchange reactions. Here we demonstrate that the so far unexplored Trx2 from African trypanosomes (*Trypanosoma brucei*) lacks protein disulfide reductase activity but functions as an effective temperature-activated and redox-regulated chaperone. Immunofluorescence microscopy and fractionated cell lysis revealed that Trx2 is located in the mitochondrion of the parasite. RNA-interference and gene knock-out approaches showed that depletion of Trx2 impairs growth of both mammalian bloodstream and insect stage procyclic parasites. Procyclic cells lacking Trx2 stop proliferation under standard culture conditions at 27°C and are unable to survive prolonged exposure to 37°C, indicating that Trx2 plays a vital role that becomes augmented under heat stress. Moreover, we found that Trx2 contributes to the *in vivo* infectivity of *T*. *brucei*. Remarkably, a Trx2 version, in which all five cysteines were replaced by serine residues, complements for the wildtype protein in conditional knock-out cells and confers parasite infectivity in the mouse model. Characterization of the recombinant protein revealed that Trx2 can coordinate an iron sulfur cluster and is highly sensitive towards spontaneous oxidation. Moreover, we discovered that both wildtype and mutant Trx2 protect other proteins against thermal aggregation and preserve their ability to refold upon return to non-stress conditions. Activation of the chaperone function of Trx2 appears to be triggered by temperature-mediated structural changes and inhibited by oxidative disulfide bond formation. Our studies indicate that Trx2 acts as a novel chaperone in the unique single mitochondrion of *T*. *brucei* and reveal a new perspective regarding the physiological function of thioredoxin-type proteins in trypanosomes.

## Author summary

African trypanosomes are the causative agents of human sleeping sickness and Nagana cattle disease. These strictly extracellular pathogens multiply in the blood and body fluids of their mammalian hosts and the tsetse fly vector, where efficient redox regulation is essential for parasite survival. While most organisms use the glutathione/glutathione reductase and thioredoxin/thioredoxin reductase couples to maintain cellular redox balance, trypanosomes rely on a unique trypanothione-based thiol metabolism to survive exogenous and endogenous oxidative stresses. Despite the lack of thioredoxin reductases, the *Trypanosoma brucei* genome encodes thioredoxins, raising questions for their biological function. Our work is the first report on *T*. *brucei* thioredoxin-2 (Trx2). We show that Trx2 is located in the mitochondrion and its absence affects parasite proliferation and infectivity. Recombinant Trx2 lacks protein disulfide reductase activity but protects proteins against aggregation and maintains them folding-competent. Remarkably, a mutant that is devoid of any cysteine residues is able to fully substitute for the authentic protein under *in vitro* and *in vivo* conditions. Our data reveal that Trx2 does not function as a classical thioredoxin but acts as a chaperone that plays a crucial role in the mitochondrion of *T*. *brucei*.

## Introduction

Trypanosomatids, the causative agents of human sleeping sickness and Nagana cattle disease in Africa (*Trypanosoma brucei* species), South-American Chagas’ disease (*T*. *cruzi*) and the different forms of leishmaniasis (*Leishmania* species), possess an unusual thiol redox metabolism. While most organisms rely on the glutathione/glutathione reductase and thioredoxin (Trx)/thioredoxin reductase systems to maintain cellular redox homeostasis, the main low molecular mass thiol in trypanosomatids is trypanothione, bis(glutathionyl) spermidine; T(SH)_2_ [1–5]. The dithiol is kept in the reduced form by the NADPH-dependent trypanothione reductase which catalyzes the only reaction directly connecting the thiol and dinucleotide redox metabolism in these parasitic protozoa. The T(SH)_2_ system provides reducing equivalents for vital processes such as the synthesis of DNA precursors and the detoxification of hydroperoxides by different types of tryparedoxin peroxidases [6–9]. An important mediator for T(SH)_2_-dependent reactions is tryparedoxin (Tpx), an essential and parasite-specific distant member of the thioredoxin (Trx)-type protein family [10].

In addition to Tpx, African trypanosomes possess conventional small oxidoreductases such as glutaredoxins [11–13] and Trxs [14]. Trx-type proteins are ubiquitous in nature. They are characterized by a common structural fold and a catalytic CXXC motif located on the surface of the protein [15, 16]. Trx was originally isolated from *Escherichia coli* as an electron donor for ribonucleotide reductase [17]. However, members of the Trx protein family have since been shown to exhibit multiple physiological functions. In addition to their role as general thiol-disulfide oxidoreductases, they can function in transcription factor regulation, protein binding, protein folding facilitation and chaperone-type activities [16]. So far, only one Trx has been characterized in *T*. *brucei*. This Trx1 (TriTrypDB gene ID Tb927.9.3370) has the canonical WCGPC motif of classical Trxs and catalyzes the electron transfer from T(SH)_2_ onto the parasite ribonucleotide reductase as well as tryparedoxin peroxidases but with much lower efficiency compared to Tpx [18]. Since thioredoxin reductases are missing in trypanosomatids, Trx1 is most likely kept reduced by thiol-disulfide exchange with T(SH)_2_. The protein does not seem to be essential at least under standard culture conditions and may be functionally replaced by Tpx [14].

African trypanosomes are obligate free-living parasites that multiply as bloodstream (BS) form in the blood and body fluids of their mammalian hosts and as procyclic (PC) insect form in the tsetse fly vector. BS *T*. *brucei* harbor a mitochondrion that lacks a cytochrome-containing electron transport chain and rely exclusively on glycolysis for energy supply. In contrast, in the PC stage, the single mitochondrion is fully elaborated and the parasites gain ATP via oxidative phosphorylation. All enzymes involved in the biosynthesis of T(SH)_2_ as well as trypanothione reductase and Tpx are located in the cytosol. The nature of the thiol metabolism in the mitochondrion of trypanosomatids is still largely unknown [19]. The primary aim of this work was to identify the missing oxidoreductase in the redox metabolism of the mitochondrion in African trypanosomes. In addition to the gene for Trx1, the genome of *T*. *brucei* encodes another putative Trx (TriTrypDB gene ID Tb927.3.4240). This previously uncharacterized Trx2 is larger than classical Trxs with a predicted molecular mass of 21.8 kDa and its amino acid sequence contains five cysteine residues, two of which form a CKPC motif. The protein is only distantly related to *T*. *brucei* Trx1 and other classical Trxs and does not appear to have any counterpart outside trypanosomatids. Different prediction algorithms (see below) suggest a mitochondrial localisation and a genome-wide RNA interference approach in *T*. *brucei* strongly suggests that the protein is essential [20].

Here we investigated the molecular properties of Trx2 both *in vitro* and *in vivo*. We show that Trx2 is located in the mitochondrion of BS and PC *T*. *brucei*. Depletion of Trx2 results in a proliferation defect in both parasite stages and PC cells virtually lacking a Trx2 species are unable to sustain long-term exposure to 37°C. However, a mutant in which all five cysteines were replaced by serine residues (5S-Trx2) can replace the authentic protein both *in vitro* and *in vivo* in the mouse model. Remarkably, recombinant Trx2 and 5S-Trx2 lack insulin reductase activity but, instead, slow down the formation of insulin aggregates. Reduced Trx2 and the 5S-Trx2 mutant prevent the aggregation of thermally unfolding proteins and preserve the folding competent state of client proteins. Activation of the chaperone function appears to be induced by conformational changes at elevated temperatures. This structural reorganization is impaired by disulfide bond formation, keeping Trx2 in a chaperone-inactive state. Taken together, we conclude that Trx2 plays a vital, redox-regulated, role in the mitochondrion of *T*. *brucei* which becomes even more important when the parasites face a long-term heat stress.

## Results

### Trx2 is an unusual thioredoxin-type protein of African trypanosomes

The protein sequence of Trx2 as deduced from the genome of the *T*. *brucei* 427 strain comprises 200 amino acid residues. It is identical to the *T*. *b*. *gambiense* ortholog and differs in just two and one position(s) from the *T*. *b*. *brucei* 927 and *T*. *evansi* sequences, respectively (S1A Fig). With only 32% overall identity, the *T*. *brucei* and *T*. *cruzi* Trx2 sequences are barely related, in striking contrast to the 74% identical Trx1 sequences from these parasites (S1B Fig). The situation in *Leishmania* is not clear. For *L*. *major* and *L*. *braziliensis*, proteins with more than 450 residues are annotated as orthologs/paralogs, whereas in *L*. *infantum* and *L*. *donovani* proteins with only 112 residues, corresponding to the C-terminal part of *T*. *brucei* Trx2, are described as putative Trxs. None of the proteins has been studied so far and future work is required, especially because the overall genomic locus is highly conserved between the various *Leishmania* species.

*T*. *brucei* Trx2 has five cysteine residues of which Cys63 and Cys66 form a CXXC motif, one of the hallmarks of Trx-type proteins [16]. The long putative *Leishmania* proteins—but not the *T*. *cruzi* ortholog—display the motif as well. The other cysteine residues are conserved only in the proteins from African trypanosomes. *T*. *brucei* Trx2 is considerably longer than classical Trxs and shares only 18% of all residues with *T*. *brucei* Trx1 which is a classical Trx and more closely related to human Trx1 [21] (S1B Fig). Structural modeling of *T*. *brucei* Trx2 (https://swissmodel.expasy.org/interactive) revealed that the protein may adopt a Trx-fold with a long central insertion (see the alignment in S1A Fig) and with the CXXC motif correctly placed at the N-terminus of an α-helix. A Blast-P search with *T*. *brucei* Trx2 against all non-redundant GenBank CDS translations retrieved exclusively Trx-like proteins but no glutaredoxins or tryparedoxins. The top hits belonged to Kinetoplastid Trx2-homologues (S1C Fig). These sequences together with those from canonical Trx proteins were subjected to multiple sequence alignment and phylogenetic tree analysis (S1D Fig). The phylogenetic analyses strongly suggest that the Trx2-like sequences from Kinetoplastida form an out-group with respect to canonical (group I) and non-canonical (group II) Trx-like proteins.

### Generation of cell lines for the *in vivo* and *in vitro* characterization of *T*. *brucei* Trx2

Table 1 summarizes the cells used in this work to evaluate the physiological role of Trx2 in BS and PC *T*. *brucei*. The molecular biology procedures are described in the Materials and methods section. Several attempts to generate homozygous *trx2* knockout (KO) cell lines were unsuccessful. PCR analysis of BS and PC resistant to both selection markers revealed that all clones had retained a *trx2* copy (S2A and S2B Fig) which strongly suggested that Trx2 is essential in both developmental stages. Therefore, we generated cKO cell lines that expressed a Tet-inducible ectopic copy of WT-Trx2 (S2C Fig). To unveil if the parasites require a redox-active form of Trx2, we produced cKO cell lines that expressed mutants in which Cys63 and Cys66 of the CXXC motif (C63/66S-Trx2) or all five cysteines (5S-Trx2) were replaced by serine residues (S2D Fig).

**Table 1:** *T*. *brucei* cell lines used in this work.

Abbreviation	Description
**WT parasites**	BS or PC *Trypanosoma brucei* that constitutively express the tetracycline repressor (cell line 449)
**2T1 Trx2**	BS 2T1 cells stably transfected with a construct for Tet-inducible expression of WT-Trx2-myc_6_
**Trx2 RNAi**	BS or PC 449 cells stably transfected with a construct for Tet-inducible RNA-interference against Trx2
**WT-Trx2 cKO**	BS or PC 449 cells in which both *trx2* alleles were replaced by resistance genes and which were stably transfected with a construct for Tet-inducible expression of WT-Trx2-myc_2_
**C63/66S-Trx2 cKO**	BS or PC 449 cells in which both *trx2* alleles were replaced and which were stably transfected with a construct for Tet-inducible expression of a Trx2-myc_2_ species in which both cysteines of the CXXC motif were replaced by serine residues
**5S-Trx2 cKO**	BS or PC 449 cells in which both *trx2* alleles were replaced and which were stably transfected with a construct for Tet-inducible expression of a Trx2-myc_2_ species in which all five cysteines were replaced by serine residues

### Trx2 localizes to the mitochondrion

**Fig 1:**
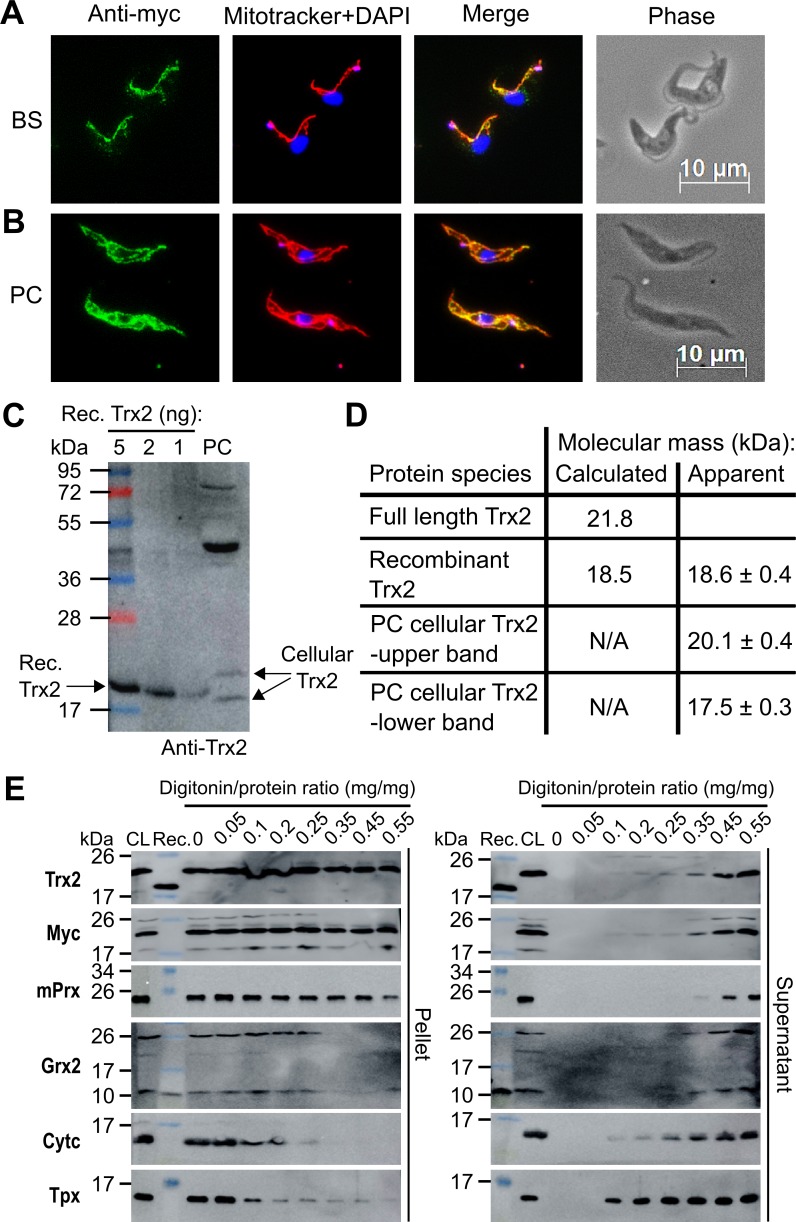
Subcellular localisation, expression level and molecular mass of mature Trx2 in *T*. *brucei*. Immunofluorescence microscopy of (**A**) BS *T*. *brucei* 2T1 cells ectopically expressing WT-Trx2 and (**B**) PC WT-Trx2 cKO cells using anti-myc antibodies (green). The mitochondrion was stained with MitoTracker (red) and the kinetoplast (small dot) and nucleus (large dot) with DAPI (blue). Merge, overlay of the three signals. Phase, phase contrast image. (**C)** Representative comparative Western blot using different amounts of recombinant (Rec.) Trx2, total lysate of 1 x 10^7^ PC *T*. *brucei* cells (PC), Page Ruler Plus for standardization and Image J for quantification. In the cell lysate, the Trx2 antibodies detected two protein species that run above and below the recombinant protein. The bands with masses > 40 kDa are proteins that cross-react with the polyclonal antiserum (see also Figs 2A and 3A). (**D**) Calculated and apparent molecular masses of the Trx2 species as derived from Western blot analyses using both PageRuler Plus and DualColor as standards. The values are the mean ± standard deviation (SD) derived from four independent analyses. (**E**) PC WT-Trx2 cKO cells were treated with increasing digitonin/protein ratios. Pellet and supernatant fractions were subjected to Western blot analyses alongside recombinant Trx2 or Grx2 (Rec.) and total cell lysate (CL). Grx2 and cytochrome c (Cytc) served as markers for the mitochondrial IMS, and Tpx and mPrx for the cytosol and the mitochondrial matrix, respectively.

The prediction algorithms MitoProt II, PredSL, PROlocalizer, TargetP1.1 and PSORT 2 suggest a mitochondrial localisation for *T*. *brucei* Trx2. To experimentally verify the subcellular localisation, BS 2T1 cells expressing WT-Trx2 and PC WT-Trx2 cKO (Table 1) were cultured in the presence of Tet and subjected to immunofluorescence microscopy (Fig 1A and 1B). The myc signals displayed a punctate pattern overlaying with the Mitotracker signal. The mitochondrial localisation of Trx2 was independently confirmed by fractionated cell lysis (Fig 1E) and is in accordance with recent proteome analyses [22, 23].

To detect the authentic protein in the parasites, antibodies against recombinant Trx2 were raised. Since the prediction algorithms described above did not provide a clear cleavage site for the mitochondrial pre-sequence, we decided to generate a recombinant Trx2 species with the 32 N-terminal residues cleaved off and an Arg at the -2 position, a characteristic of the cleavage sites of mitochondrial processing peptidase (MPP [24, 25]) (S1A Fig). The molecular mass of recombinant Trx2 derived from Western blot analysis (18.55 ± 0.4 kDa) was in agreement with its calculated mass of 18,467 Da (-32 residues, plus an additional N-terminal GAMG stretch due to the TEV-cleavage). In cell lysates of *T*. *brucei*, the antibodies consistently detected a doublet of bands that migrated slightly above and below the recombinant protein (Fig 1C). Both bands were diminished/absent when the cells were subjected to Trx2 mRNA depletion and re-appeared when the cells had lost the RNAi regulation (see below). The apparent average molecular masses of the bands were 20.1 and 17.5 kDa (Fig 1D). As full-length Trx2 has a theoretical mass of 21.82 kDa and the recombinant full-length protein runs at a much higher mass than the truncated Trx2 (see S6B Fig), we concluded that both species detected in the cells are processed forms.

To evaluate the intra-mitochondrial localisation of Trx2 in more detail, PC WT-Trx2 cKO cells were treated with increasing concentrations of digitonin, which results in the gradual permeabilization of cellular membranes [26]. The pellet and supernatant fractions were subjected to Western blot analysis. Antibodies against Trx2 or the myc-tag revealed a prominent band and a very weak band with slightly higher mass (Fig 1E). Up to the highest digitonin:protein ratio (0.55:1 mg/mg) applied, both Trx2 bands remained largely in the pellet fraction. The same behavior was observed for the mitochondrial 2-Cys-peroxiredoxin (mPrx), used as marker protein for the mitochondrial matrix. At digitonin:protein ratios of ≥ 0.35:1, Trx2 and mPrx became only partially solubilized whereas the intermembrane space proteins Grx2 and Cyt c as well the cytosolic Tpx were virtually completely in the supernatant fractions. Thus, we conclude that the two Trx2 bands detected in the Western blots represent the partially and fully processed protein in the mitochondrial matrix.

To estimate the cellular concentration of Trx2, total lysates of PC *T*. *brucei* and different amounts of the recombinant protein were studied by comparative Western blot analyses. Based on the data from seven independent analyses and using 96 fl as volume of PC cells [27], we calculated a cellular concentration of Trx2 of 131 ± 49 nM. The mitochondrion of PC *T*. *brucei* has been reported to occupy about one fourth of the total cell volume [28]. Thus, the local concentration of Trx2 in the mitochondrion would be in the order of 0.5 μM.

### Depletion of Trx2 affects *in vitro* proliferation and impairs parasite survival *in vivo*

**Fig 2:**
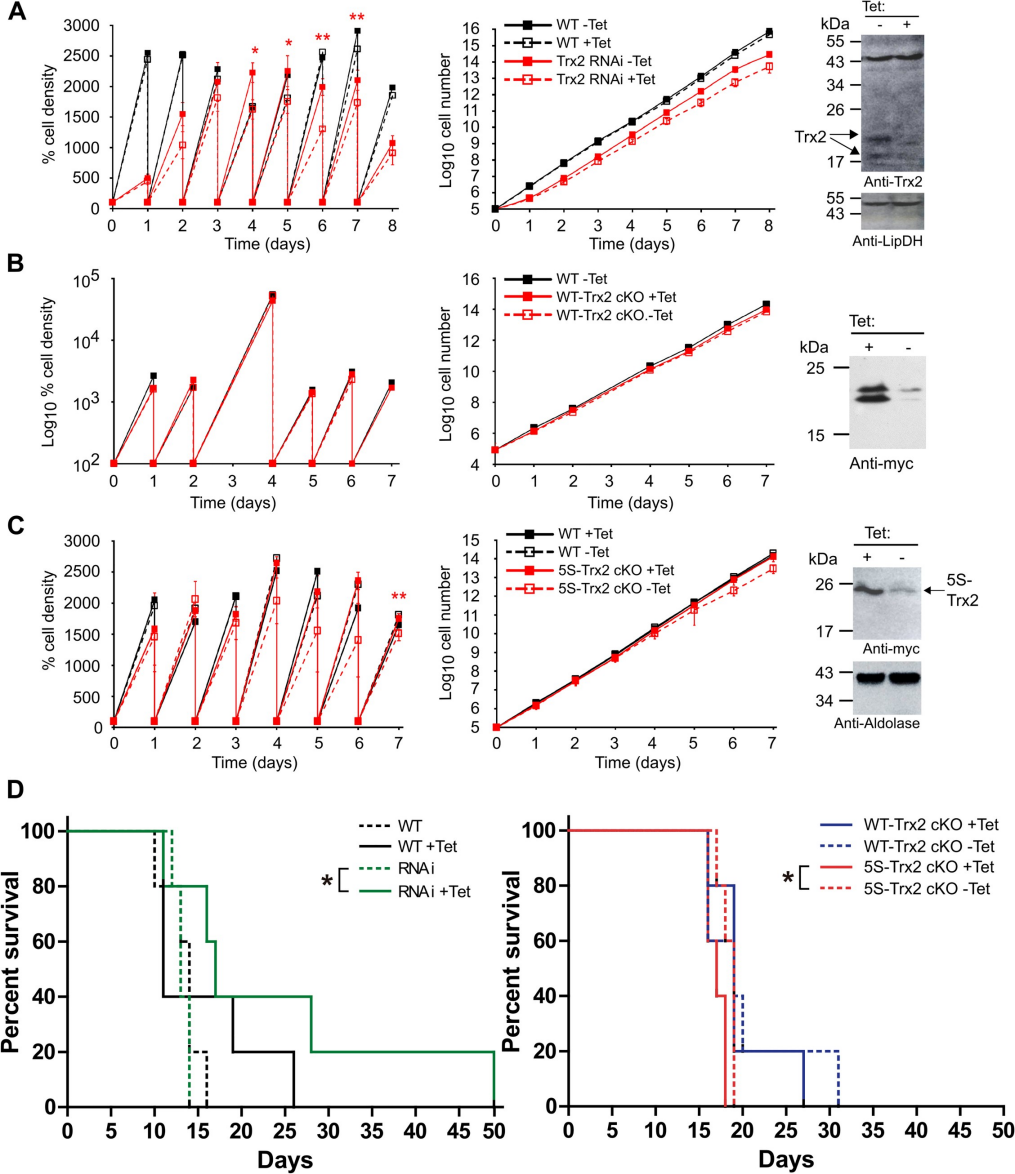
*In vitro* and *in vivo* analysis of Trx2 in BS *T*. *brucei*. (**A**) Cells harboring a construct for tet-inducible RNAi against Trx2, (**B**) WT-Trx2 cKO cells and (**C**) 5S-Trx2 cKO cells as well as WT parasites were cultured in the presence or absence of 1 μg/ml Tet. Every 24 h, the cells were counted and diluted back to the starting density of 1 x 10^5^ cells/ml. The left graphs depict (**A** and **C**) the percentage of cells relative to the starting density set as 100% (mean ± SD) from three distinct cell lines and (**B**) the increase in cell density after each 24 h time point. The right graphs show the cumulative cell densities (mean ± SD). All analyses were conducted in duplicate. A paired t-test was used to evaluate significant differences between (**A**) non-induced and induced RNAi cell lines and (**C**) 5S-Trx2 cKO cells grown in the presence or absence of Tet at each time point (* = p<0.05 and ** = p<0.01). Western blot analyses are shown for total lysates from (**A**) 2 x 10^7^ Trx2 RNAi cells grown in the absence (-) or for five days in the presence of Tet (+) and lipoamide dehydrogenase (LipDH) as loading control, (**B**) 1 x 10^7^ WT-cKO cells grown in the presence (+) or for four days in the absence (-) of Tet and (**C**) 1 x 10^7^ 5S-Trx2 cKO cells cultured in the presence (+) or for five days in the absence (-) of Tet and aldolase as loading control. (**D**) Groups of five animals fed with (+Tet) or without oxytetracycline in the drinking water were infected with 10^4^ WT parasites, a Tet-inducible Trx2 RNAi cell line (RNAi) or Tet-inducible cKO cell lines expressing either WT-Trx2 or 5S-Trx2. The Kaplan Meier plots show the survival for each group of mice infected with (left) WT parasites or the RNAi cell line, and (right) WT-Trx2 cKO or 5S-Trx2 cKO. The asterisks denote statistical significance (Log-rank test) with *p* values of 0.044 for the compared groups.

A genome-wide RNAi library screen suggested that Trx2 is essential for *T*. *brucei* [20]. BS Trx2 RNAi cells (Table 1) as well as WT parasites were cultured in the presence or absence of Tet. Whereas proliferation of WT parasites was unaffected by Tet, the induced Trx2 RNAi cell lines displayed a minor but significant growth retardation between day 4 and 7, when compared to the respective non-induced cells (Fig 2A). For example, between day 5 and 6, WT parasites, non-induced and induced RNAi cells multiplied by a factor of 25, 20 and 12, respectively. Western blot analyses of cells harvested after five days of Tet exposure revealed a partial depletion of the protein. Apparently, the remaining comparably low level of Trx2 still allowed the parasites to proliferate.

Cultivation of BS WT-Trx2 cKO cells (Table 1) in Tet-free medium for seven days did not affect proliferation (Fig 2B). Western blot analyses confirmed that the cells, albeit at significantly lower levels, still expressed the ectopic copy of Trx2. As tightly regulated RNAi or WT-Trx2 cKO cell lines could not be obtained, we asked if the parasites require a redox-competent form of the protein. In the presence of Tet, C63/66S-Trx2 cKO cell lines (Table 1) were viable and proliferative (S3A Fig). In the absence of Tet, the cells showed a minor and only transient growth defect. Finally we generated BS cells that harbored solely a cysteine-free mutant of the Trx2. Tet-removal from cultures of these 5S-Trx2 cKO cells (Table 1) also resulted in only a very minor proliferation defect. Again low levels of the protein were still detectable after five days in Tet-free medium (Fig 2C).

In order to assess the biological relevance of Trx2 for parasite proliferation and survival in a mammalian host, mice (five per group) were infected with BS *T*. *brucei* cell lines that allowed the Tet-inducible downregulation of the endogenous protein (Trx2 RNAi) or expression of a myc-tagged ectopic copy of WT-Trx2 (WT-Trx2 cKO) or 5S-Trx2 (5S-Trx2 cKO) in a Trx2 KO genetic background (see Table 1 for the different cell lines). Animals infected with WT *T*. *brucei* served as controls. Parasitemia and animal survival were monitored (S4 Fig and Fig 2D). In agreement with our earlier studies [27, 29], feeding the animals with water containing oxytetracycline (1 mg/ml, +Tet) did not have any effect on the outcome of the infection for mice challenged with WT parasites (S4 Fig and Fig 2D; *p* values >> 0.05 for parasitemia and survival).

**Fig 3:**
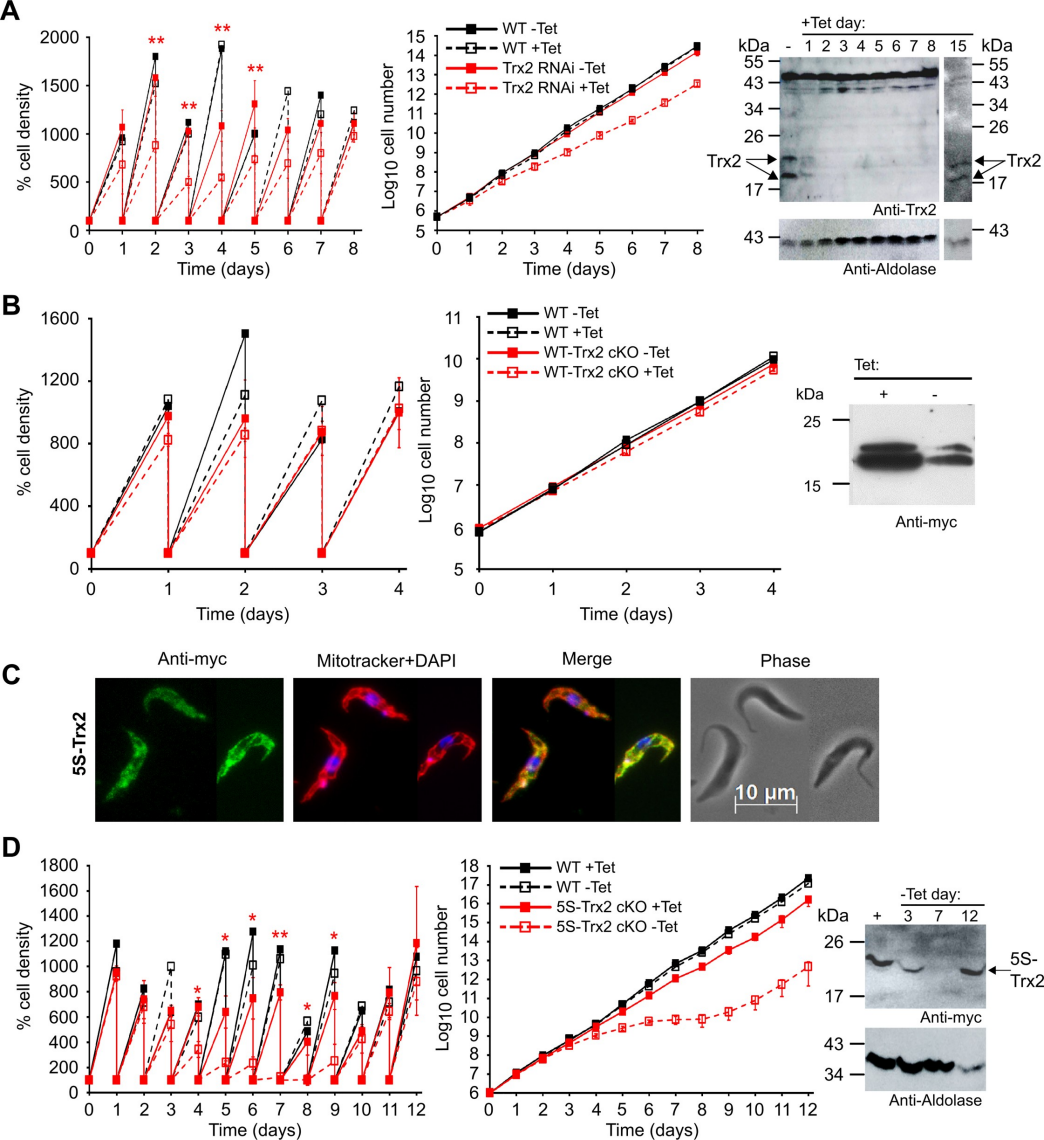
Analysis of Trx2 in PC *T*. *brucei*. Tet-inducible (**A**) Trx2 RNAi, (**B**) WT-Trx2 cKO and (**D**) 5S-Trx2 cKO cell lines as well as WT parasites were cultured ± Tet. Every 24 h, the cells were counted and diluted back to the starting density of 5 x 10^5^ cells/ml and analyzed as outlined in the legend of Fig 2. The data are the mean standard ± SD from three distinct cell lines. All analyses were conducted in duplicate. A paired t-test was used to evaluate significant differences between non-induced and induced cell lines at each time point (* = p<0.05 and ** = p<0.01). Western blot analyses were conducted with total lysates from 1 x 10^7^ (**A**) Trx2-RNAi cells grown in the absence or for different times in the presence of Tet, (**B**) WT-Trx2 cKO cells grown in the presence or for four days in the absence of Tet and (**D**) 5S-Trx2 cKO cells cultured in the presence of Tet or for different times in the absence of Tet. Aldolase served as loading control. (**C**) Immunofluorescence microscopy of induced 5S-Trx2 cKO cells using anti-myc antibodies (green). The mitochondrion was stained with MitoTrackerRed (red) and the kinetoplast and nucleus with DAPI (blue). Merge, overlay of the three signals. Phase, phase contrast image.

As observed in previous *in vivo* studies [13, 29, 30], the parasitemia dropped to almost undetectable levels about one week post infection (S4 Fig, day 6), a phenomenon associated with the acute host immune response aimed to control infection [31]. The survival of +Tet mice infected with Trx2 RNAi parasites was significantly extended when compared to the animals where Trx2 expression was not silenced (Fig 2D; *p* = 0.044). In fact, animals from the non-induced RNAi group died between day 11 and 14 post infection, similar to those infected with WT parasites. In comparison, the fatal outcomes in mice of the RNAi +Tet group occurred between day 11 and 49 post infection. The median survival time was estimated to be 13 and 17 days for the non-induced and induced RNAi-group, respectively. Despite the prolonged survival, all mice from the +Tet RNAi group died. This can be ascribed to the stochastic appearance *in vivo* (and also *in vitro*, Fig 3) of mutant parasites that are refractory to the RNAi silencing. The phenomenon is often observed for essential proteins in *T*. *brucei* [32–34]. Although statistically not significant, likely due to the low animal number from day 9 onwards, the median parasitemia was consistently lower in mice from the +Tet group compared to the non-induced RNAi group (S4 Fig, day 9, 11, and 13). As shown in Fig 2A, RNAi partially depleted Trx2 in BS cells which resulted in a minor but significant *in vitro* proliferation defect. Assuming a similar RNAi efficiency under *in vivo* conditions, low levels of Trx2 are probably sufficient for the parasite to establish host infection. Nonetheless, the sustained lower parasite load in +Tet mice (S4 Fig) suggests an impaired capacity of the pathogen to proliferate in the host when Trx2 is downregulated, and correlates well with the longer animal survival time exhibited by this group (Fig 2D, left).

As shown in Fig 2B and 2C, after 4 or 5 days of cultivation in the absence of Tet, the WT-Trx2 and 5S-Trx2 cKO cell lines still expressed Trx2. Thus, as in the RNAi approach, this system did not allow to generate cells that lacked the protein completely. Yet, the cKO cell lines offered the possibility to address a potential redox role of Trx2 as a virulence or signaling factor *in vivo*. Both cKO cell lines were able to establish mouse infection, which displayed a similar progress independently whether the animals were fed with oxytetracycline to sustain the expression of the ectopic Trx2 species or not (Fig 2D, right). Notably, under +Tet and -Tet conditions, the cKO cell lines displayed a significantly (*p* values < 0.05) lower virulence (median survival time from 17 to 19 days) than WT parasites +Tet (median survival time of 11 days) or the non-/induced RNAi cell line (14 days). Maybe the genetic manipulations required to generate the cKO cells affect their *in vivo* virulence or the C-terminal tag slightly interferes with the physiological role(s) of the protein during *in vivo* infection. Animals infected with WT-Trx2 cKO cells displayed an identical median survival time of 19 days for the + and -Tet groups (*p* value = 0.759) and no differences in parasitemia were observed (S4 Fig). In contrast, mice infected with the 5S-Trx2 cKO cells and fed with oxytetracycline showed a mean survival time (17 days) that was significantly shorter (p value = 0.044) than that of the -Tet group (19 days). This could not be ascribed to differences in parasite load because statistical analysis did not yield significant results (p value >> 0.05).

In summary, down-regulation of Trx2 impairs development of *T*. *brucei* in a mammalian host and thus shows that Trx2 is required for full infectivity under *in vivo* conditions. Strikingly, cKO parasites that exclusively expressed 5S-Trx2 were at least as infectious as those expressing an ectopic copy of WT-Trx2. Thus, the *in vivo* Trx2 functions also do not involve thiol-redox reactions.

### A Trx2 species is essential for the proliferation of PC *T*. *brucei*

Depletion of Trx2 in PC cell lines caused a significant growth defect (Fig 3A). In Western blots, the protein was no longer detectable 48 h after RNAi induction. Upon prolonged cultivation, the PC Trx2 RNAi cell lines (Table 1) resumed proliferation despite the presence of Tet which was accompanied by re-appearance of the protein. This phenomenon is often observed for essential *T*. *brucei* proteins and implies that the parasites managed to escape from the inducible RNAi system [33, 35].

Cultivation of three distinct PC WT-Trx2 cKO cell lines (Table 1) for four days in Tet-free medium did not affect proliferation (Fig 3B). Also C63/66S-Trx2 cKO cell lines showed only a minor growth defect when transferred into medium without Tet (S3B Fig) and Western blot analyses of cells that were kept for five days in Tet-free medium still revealed the presence of the protein (S3C Fig). Immunofluorescence microscopy showed that the ectopically expressed C63/66S-Trx2 was correctly targeted to the mitochondrion (S3D Fig). Finally, we generated PC cell lines that harboured a cysteine-free mutant as sole Trx2 species (Table 1). Immunofluorescence microscopy confirmed the specific targeting of the protein into the mitochondrion (Fig 3C). In contrast to the respective BS mutants, PC 5S-Trx2 cKO cells lines stopped proliferation about four days after Tet removal and at day 7, the protein was almost undetectable (Fig 3D). Upon continuous cultivation in Tet-free medium, the cells resumed normal growth. Reappearance of 5S-Trx2 at a level comparable to that in the constantly induced control cells at day 12 indicates that regulation of the Tet-inducible system was lost. In summary, our data show that Trx2 is required for the proliferation of both BS and PC *T*. *brucei*, and demonstrate that a Trx2 species that is devoid of any cysteine residue is sufficient to maintain cell viability and proliferation *in vitro*.

### Sensitivity of WT and cKO cells towards exogenous reductive and oxidative stressors

In a transcriptome analysis of PC *T*. *brucei* that were treated for 1 or 3 h with 4 mM DTT in order to induce an ER stress response, the Trx2 mRNA level was reported to be 2-fold increased [36]. Due to the known communication between mitochondria and the ER in eukaryotic cells [37], we evaluated if the higher transcript level correlates with an increased protein concentration. For this purpose, we treated WT parasites with 4 mM DTT and subjected the total lysates of stressed and non-stressed cells to Western blot analysis. No significant difference in the Trx2 protein level was observed (S5A Fig). Subsequently, WT and induced 5S-Trx2 cKO cells (Table 1) were treated with DTT and cell viability was followed. Under our experimental settings, PC *T*. *brucei* were highly sensitive towards DTT in accordance with a previous study [38]. In the presence of 150 μM DTT, the parasites started to die within 2 h, and after 8 h, less than 50% of cells were still viable (S5B Fig). Under all conditions studied, WT and 5S-Trx2 cKO cells displayed identical sensitivity.

Whereas in most organisms peroxiredoxins (Prxs) use Trxs as an electron source, the known trypanosomatid 2-Cys-peroxiredoxins are Tpx-dependent [19, 39, 40]. The parasite Prxs occur in the cytosol and mitochondrion, but Tpx appears to be restricted to the cytosol [39]. To get an insight if the mitochondrial Trx2 could play a role in hydroperoxide detoxification, we treated WT parasites and 5S-Trx2 cells with H_2_O_2_. Both cell types showed a very similar behavior when exposed to short- or long-term peroxide stress (S5D Fig). Paraquat is a redox-cycling compound that generates superoxide anions which are converted into hydrogen peroxide, and the mitochondrion is a main compartment of its mode of action [41, 42]. At all time-points studied and both concentrations applied, paraquat affected the proliferation of WT parasites, WT-Trx2 cKO and 5S-Trx2 cKO cells (Table 1) to the same degree (S5D Fig). In addition, cKO cells that were grown for five days in the absence of Tet and thus had strongly reduced levels of the protein (see Fig 3B and 3D), did not reveal an increased sensitivity. Taken together, Trx2 does not seem to play a role in the response of *T*. *brucei* to exogenous reductive or oxidative stresses.

### Authentic or 5S-Trx2 is required for *T*. *brucei* PC cells to sustain long-term heat stress

**Fig 4:**
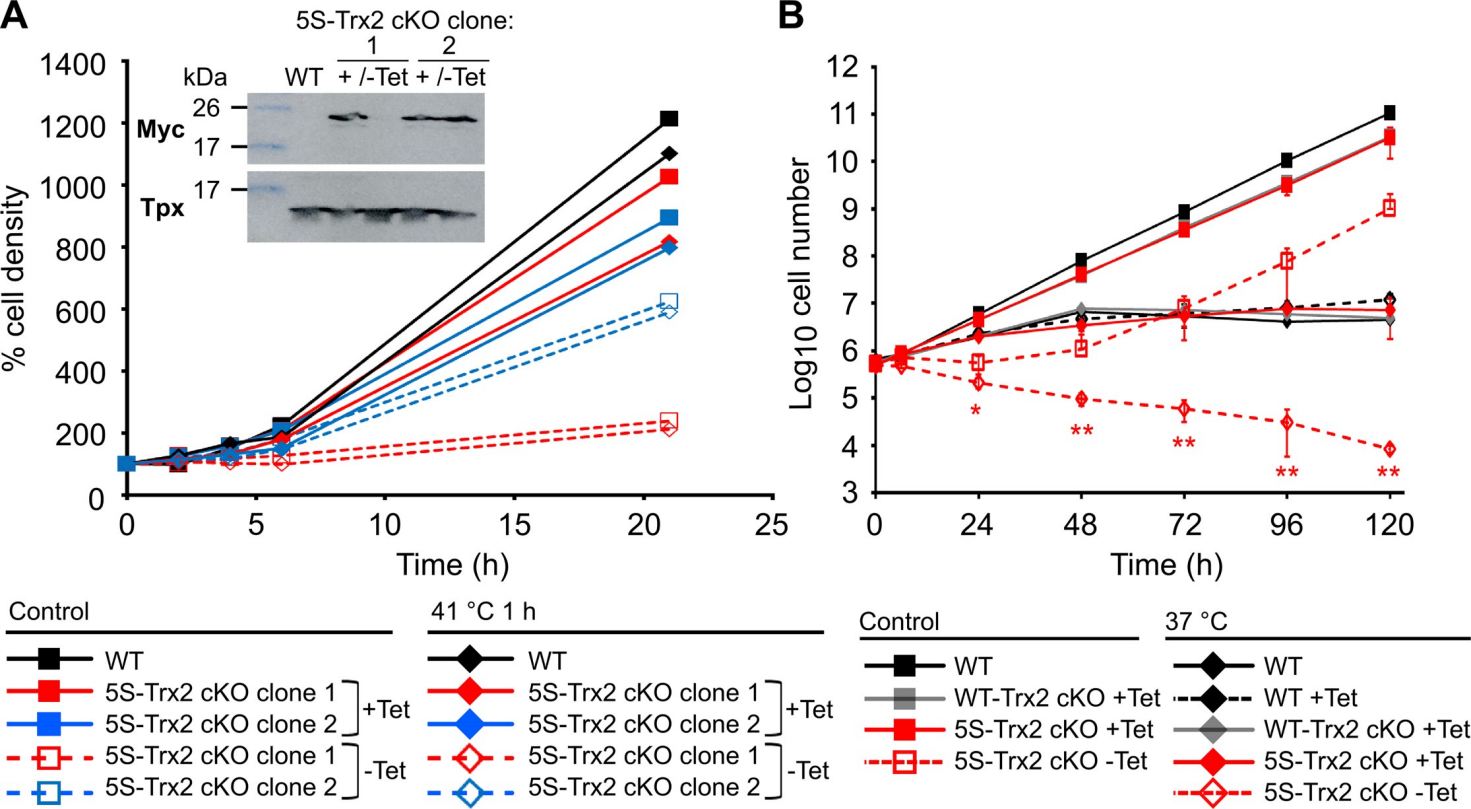
The presence of a Trx2 species is required for long term survival of PC cells at 37°C. (**A**) 5S-Trx2 cKO were cultured for five days in the absence of Tet (-Tet). Subsequently, 5S-Trx2 cKO ± Tet as well as WT parasites were either further kept under standard conditions at 27°C (control) or incubated for 1 h in 41°C pre-heated medium and then re-transferred to 27°C. After different times, living cells were counted. Inset, Western blot analysis of lysates from 2 x 10^7^ WT parasites and two 5S-Trx2 cell lines grown for five days ± Tet with antibodies against the myc-tag and Tpx as loading control. Clone 1 showed a severe growth defect in accordance with the virtually undetectable level of the protein in the cell lysate whereas clone 2 had already resumed proliferation and again expressed Trx2. The data depicted are representative for three independent experiments conducted with three 5S-Trx2 clones. (**B**) 5S-Trx2 cKO cells were pre-cultured in Tet-free medium until they demonstrated a proliferation defect, usually observed around six days after Tet removal (see Fig 3D). WT parasites and cKO cells expressing WT-Trx2 or 5S-Trx2 as well as 5S-Trx2 cKO cells kept in Tet-free medium were either continuously grown under standard conditions (control) or transferred to 37°C. To rule out any effect of Tet on the parasites proliferation at the elevated temperature, WT cells in the presence of Tet were included as an additional control. After different times, the cells were counted and the cultures diluted to the starting density of 5 x 10^5^ cells/ml, if necessary. For the cKO cells, the data represent the mean ± SD from three cell lines. In the case of WT cells, a representative analysis of three repetitions is depicted. A paired t-test was used to evaluate significant differences between the 5S-Trx2 cKO cells grown in the absence of Tet at 27°C and 37°C, respectively (* = p<0.05 and ** = p<0.01).

As PC cells displayed a clearer proliferation defect upon down-regulation of Trx2 and the Tet-inducible expression was more tightly regulated in PC 5S-Trx2 cKO clones than in BS cells, we decided to use the insect form with its fully elaborated mitochondrion to study a putative role of Trx2 in the heat response of the parasites. In the first approach, 5S-Trx2 cKO cells that were grown for five days in the absence of Tet as well as 5S-Trx2 cKO constantly cultured in the presence of Tet and WT parasites were exposed to a 41°C heat shock for 1 h, re-transferred to 27°C and cell viability/proliferation was followed. WT parasites and the 5S-Trx2 cKO cells +Tet readily recovered from the stress. One of the 5S-Trx2 cell lines -Tet depicted in Fig 4A, had a strong proliferation defect and the protein was practically undetectable. The other clone had already resumed growth in accordance with the reappearance of the protein. The 1 h 41°C treatment had no significant additional effect on the viability of both cell lines, strongly suggesting that Trx2 does not play a significant role in the recovery of procyclic cells from a short-term heat stress (Fig 4A).

Next, we monitored the long-term proliferation of WT-Trx2 cKO and 5S-Trx2 cKO cell lines and WT parasites at 37°C (Fig 4B). Under standard conditions of 27°C, WT parasites multiplied by a factor of 10–13 every 24 h, and the induced WT-Trx2 cKO and 5S-Trx2 cKO cell lines by a factor of 8–9. At 37°C, WT parasites ± Tet and the induced cKO cells multiplied about 4-fold within the first 24 h, and 2-3-fold within the next 24 h. After 48 h exposure to 37°C, proliferation of the cells stopped until the experiment was terminated after 120 h. The response of WT parasites (± Tet) and the induced cKO cell lines to 37°C heat stress was practically identical. In contrast, 5S-Trx2 cKO cells that had been pre-cultured for 6 to 9 days in the absence of Tet (and thus lacked virtually any Trx2 (see Fig 3D) were significantly more sensitive to heat stress when compared to the induced cells (Fig 4B). Within 24 and 48 h, the cell density dropped to 43% and 20% of the starting cell number, and less than 2% of the cells were still living after 120 h. In contrast to the phenotype observed at 27°C, 5S-Trx2 cKO cells grown in the absence of Tet at 37°C did not resume growth, indicating that they were unable to overcome the Tet-regulation. The data showed that PC cells require Trx2 to withstand a prolonged 37°C heat stress but a cysteine-free form of Trx2 is able to replace the authentic protein.

### Recombinant Trx2 can coordinate an iron sulfur cluster and undergoes thiol-dependent changes in its oligomeric state

*T*. *brucei* Trx2, C63/66S-Trx2, and 5S-Trx2, lacking the 32 N-terminal residues as well as full length Trx2 were expressed as TEV-cleavable fusion proteins in *E*. *coli*. The tag-free proteins were obtained by metal affinity chromatography as outlined in Materials and methods and their purity was verified by SDS-PAGE (S6 Fig). Because the recombinant full-length protein was unstable and constantly precipitated it was not further characterized.

Freshly prepared recombinant Trx2 yielded 0.5–1.5 thiols/protein in the DTNB assay, independent of the presence or absence of 8 M urea. After treatment with TCEP or DTT, five thiol groups were detected in accordance with all cysteine residues being accessible and present in reduced form. Upon storage in the absence of a reducing agent, free thiols virtually completely disappeared. This sensitivity to spontaneous oxidation could be visualized upon SDS-PAGE. Under reducing conditions, Trx2 migrated at about 19 kDa corresponding to its theoretical mass. Under non-reducing conditions, the protein showed several bands with higher masses and, remarkably, the monomer was shifted to an apparently lower mass indicating that Trx2 formed inter- and intramolecular disulfides (S6C Fig). The C63/66S-Trx2 mutant showed a similar behavior suggesting that the cysteines that cause the shift of the oxidized monomer are distinct from those of the CXXC motif. As expected, the 5S-mutant migrated at the theoretical mass of the monomer, independent of the presence or absence of DTT.

**Fig 5:**
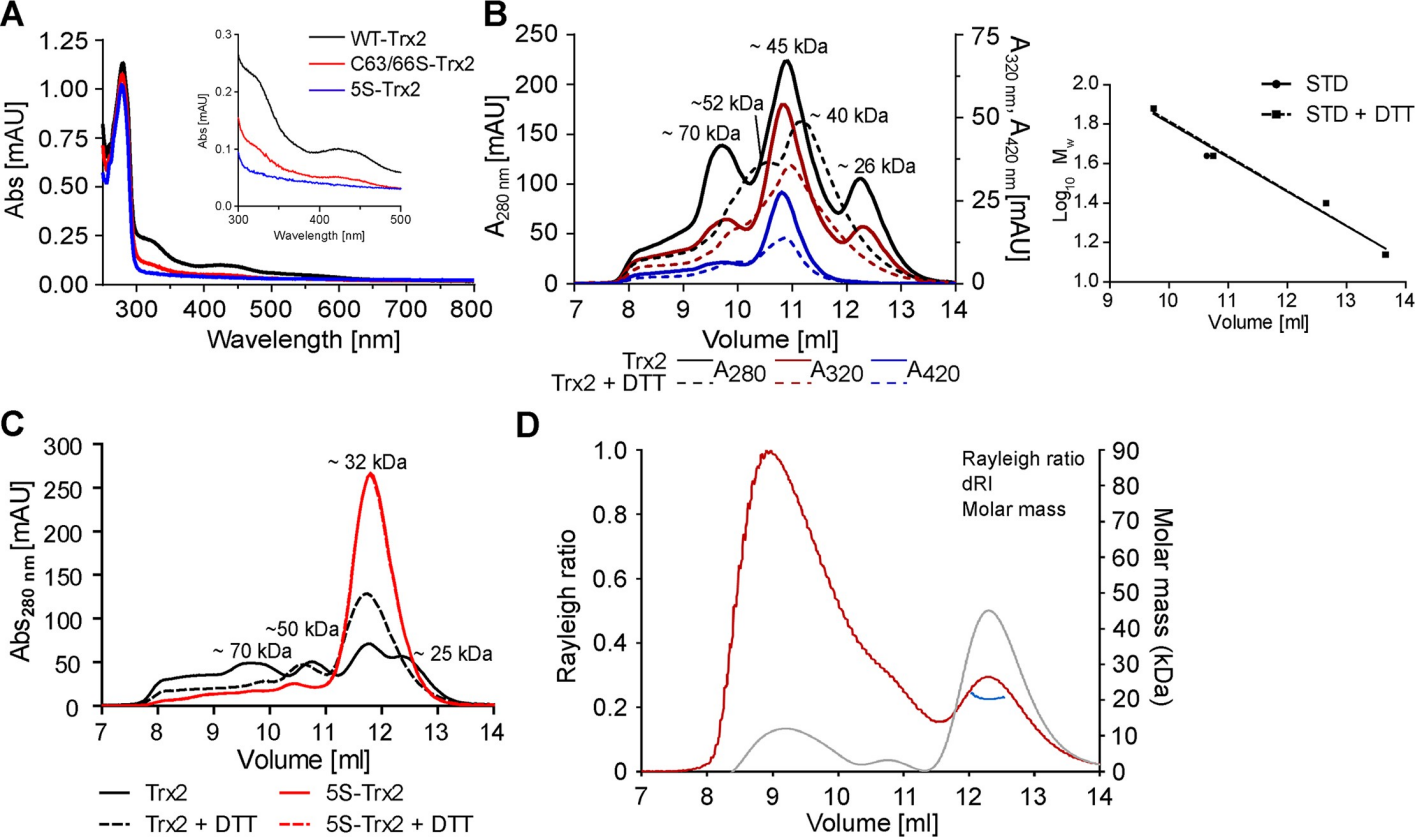
Spectroscopic properties and oligomeric state of recombinant Trx2 species. (**A**) The UV-visible spectra of 120 μM Trx2, C63/66S-Trx2 and 5S-Trx2 in 50 mM sodium phosphate, 300 mM sodium chloride, pH 8.0. (**B** and **C**) The gel filtrations were performed in 50 mM sodium phosphate, 150 sodium chloride, pH 7.0 on a Superdex 75 10/300 GL column. Trx2 and 5S-Trx2 were incubated for 30 min at 25°C with and without 25 mM DTT, centrifuged for 45 min at 13,000 rpm and 4°C, and the protein concentration in the cleared supernatant was determined. (**B**) 25 μl of pre-reduced and 50 μl of untreated Trx2 (1.2 mM), respectively, was loaded onto the column which was run at room temperature and a flow rate of 0.3 ml/min in the presence or absence of 1 mM DTT. The elution profile was recorded at 280, 320, and 420 nm. **(C)** 25 μl of untreated and pre-reduced Trx2 (0.88 mM) and 50 μl of 5S-Trx2 (0.43 mM) pre-incubated ± DTT were processed as outlined above. Protein elution was followed at 280 nm. The apparent molecular masses of the peaks were calculated from standard chromatograms. **(D)** 100 μl of 80 μM 5S-Trx2 was subjected to size exclusion chromatography with online multi-angle light scattering (SEC-MALS). Relative intensity of Rayleigh ratio (red) and differential refractive index (dRI, grey) from the light scattering detector are shown against the elution volume. The determined mass (blue) is shown for the second peak.

Recombinant Trx2 displayed a brownish color. The UV-visible spectrum revealed, in addition to the maximum at 280 nm, absorption peaks at 320 and 420 nm and resembled that of other Trx family proteins found to coordinate iron sulfur clusters [43–45]. The C63/66S-Trx2 variant had very low absorption at 320 and 420 nm which did not allow us to decide if the mutant is still able to coordinate a cluster. Clearly, 5S-Trx2 showed an absorption maximum at 278 nm but no absorption at higher wavelengths (Fig 5A). To get a deeper insight into the putative iron sulfur cluster complex, freshly prepared Trx2 was subjected to gel filtration and the absorption recorded at 280, 320, and 420 nm. The 280 nm profile revealed peaks at about 26, 45, and 70 kDa (Fig 5B, solid black line) which probably represent monomeric, dimeric and oligomeric forms of Trx2. The 26 kDa peak had absorption at 320 nm (solid red line) which might be due to some iron binding [46]. The absence of any absorption at 420 nm (solid blue line) indicates that the monomer does not bind an iron sulfur cluster. The 45 and 70 kDa peaks showed absorption at all three wavelengths suggesting that dimeric and oligomer forms of Trx2 can coordinate an iron sulfur cluster. When Trx2 was pre-reduced and the column run in the presence of DTT, the 280 nm profile displayed two broad peaks with maxima around 40 and 52 kDa (black dashed line). Interestingly, the 320 and 420 nm maxima (red and blue dashed lines) did not overlay with the 280 nm maximum but remained virtually at the position observed under non-reducing conditions. The Trx2 species with bound iron sulfur cluster appears to be unaffected by DTT.

Next, we compared the oligomeric state of Trx2 and 5S-Trx2. Under non-reducing conditions, the elution pattern of Trx2 displayed peaks at 25, 32, 50, and 70 kDa which are supposed to reflect the monomer with intramolecular disulfide, fully reduced monomer, various dimeric and oligomer forms, respectively (Fig 5C, black solid line). The 45 kDa peak seen in Fig 5B was resolved into two peaks, maybe because an only minor fraction of Trx2 was still in complex with an iron sulfur cluster or because less protein was applied onto the column. When pretreated with DTT, Trx2 eluted in one prominent peak with an apparent mass of 32 kDa (Fig 5C, black dashed lines). 5S-Trx2 eluted in a single peak independent of the presence or absence of DTT (Fig 5C, red lines). Both 5S-Trx2 and reduced Trx2 displayed an apparent mass of about 32 kDa in comparison to their theoretical masses of 18.5 and 18.4 kDa, respectively. To verify that these species are indeed monomeric forms of the protein, 5S-Trx2 was subjected to size-exclusion chromatography with multi-angle light scattering (SEC-MALS). The analysis yielded a molecular mass of 20,730 Da (Fig 5D) and confirmed that the 32 kDa peak contains monomeric 5S-Trx2. The relative intensity of the Rayleigh ratio indicated the presence of a high molecular mass species. However, the differential refractive index (dRI) showed that the concentration of this species is low in comparison to that of the monomeric form. Taken together, our data revealed that recombinant *T*. *brucei* Trx2 can assume a variety of forms such as the monomeric protein with and without intramolecular disulfides, dimers between reduced and oxidized subunits as well as covalent dimers and finally dimers and oligomers with bound iron sulfur cluster.

### Trx2 lacks insulin reductase activity

**Fig 6:**
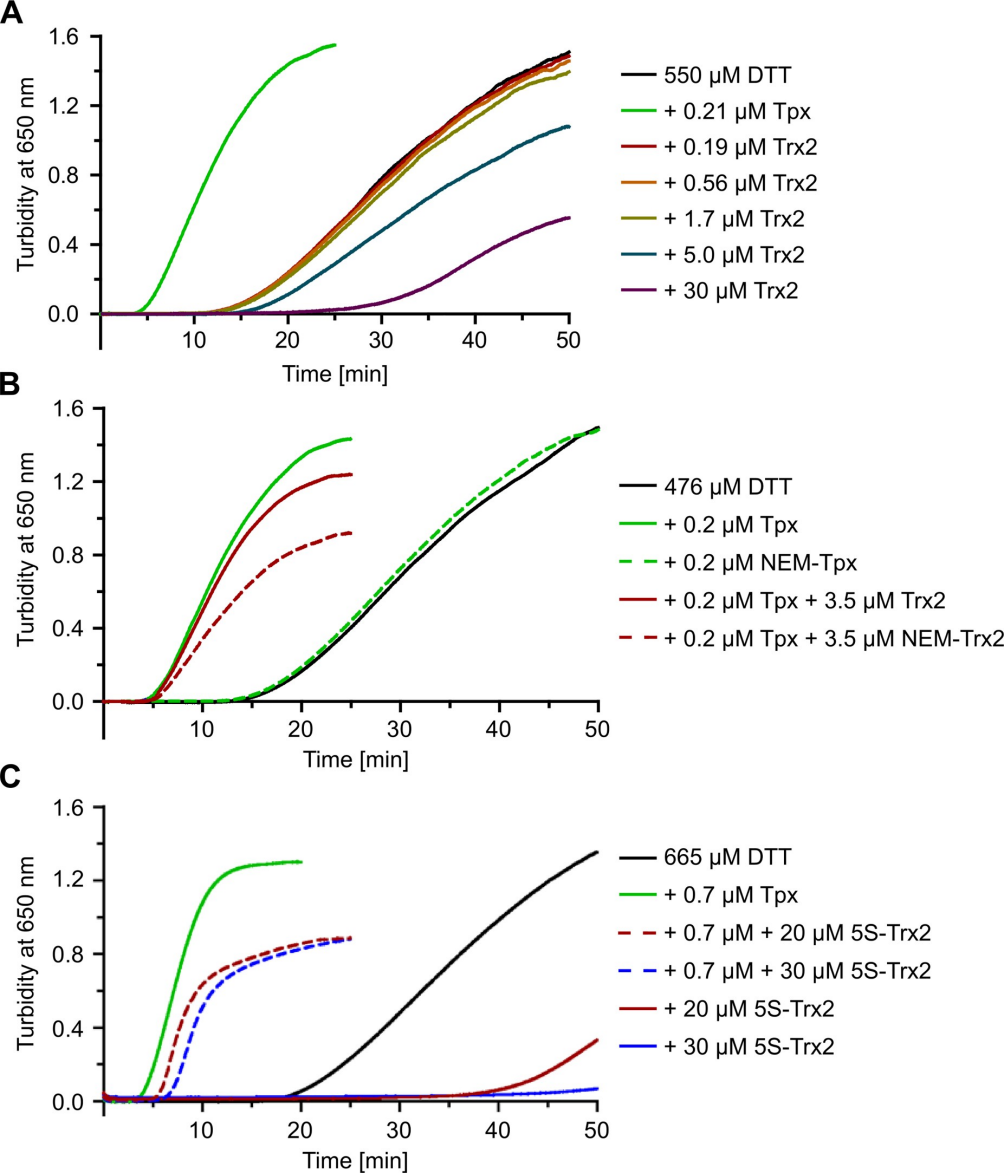
Trx2 does not catalyze insulin reduction but displays redox-independent chaperone-like activity. Reduction of insulin (130 μM) by DTT was followed in the presence of different Trx2 species. Precipitation of the insoluble B-chain of insulin was monitored at 37°C by the increase in turbidity at 650 nm. Reaction mixtures containing DTT alone (black line) or plus Tpx (green line) served as control for the spontaneous and Tpx-catalyzed reaction, respectively. The assays contained (**A**) Tpx or increasing concentrations of Trx2, (**B**) Tpx and Trx2 with or without prior alkylation with N-ethylmaleimide (NEM) and (**C**) 5S-Trx2 in the presence or absence of Tpx. The analyses in (**B**) and (**C**) were conducted at least in duplicate and representative assays are presented.

Most Trxs are known for their high protein disulfide reductase activity [15, 16]. Insulin reduction is the most convenient assay to measure the activity *in vitro*. Cleavage of the inter-chain disulfide bridges results in the release of the insoluble free B-chain which can be monitored by an increase in turbidity at 650 nm [47]. We used *T*. *brucei* Tpx as positive control [26]. Indeed, presence of 0.21 μM Tpx strongly accelerated the reduction of insulin by DTT and hence the aggregation process (Fig 6). In contrast, the presence of up to 2 μM Trx2 had no effect on the B-chain aggregation, and higher Trx2 concentrations slowed down the process (Fig 6A). These results strongly suggested that *T*. *brucei* Trx2 is not acting as protein disulfide reductase but instead might function as a molecular chaperone that protects the B-chain against irreversible aggregation.

To get a deeper insight in the underlying mechanism, Tpx and Trx2 were treated with DTT and all cysteine residues were alkylated by NEM. As expected, NEM-treated Tpx completely lost its insulin reductase activity (Fig 6B). The alkylated Trx2, on the other hand, maintained its anti-aggregation activity, and seemed to be even more effective than the non-modified protein (Fig 6B). To corroborate that the putative chaperone activity of Trx2 is really independent of the cysteine thiols, we studied insulin reduction in the presence of 5S-Trx2. Indeed, this mutant was even more effective than NEM-treated WT Trx2 in slowing down the Tpx-catalyzed reaction and almost completely prevented the spontaneous turbidity development when present at 30 μM (Fig 6C).

### Reduced Trx2 prevents protein aggregation *in vitro*

**Fig 7:**
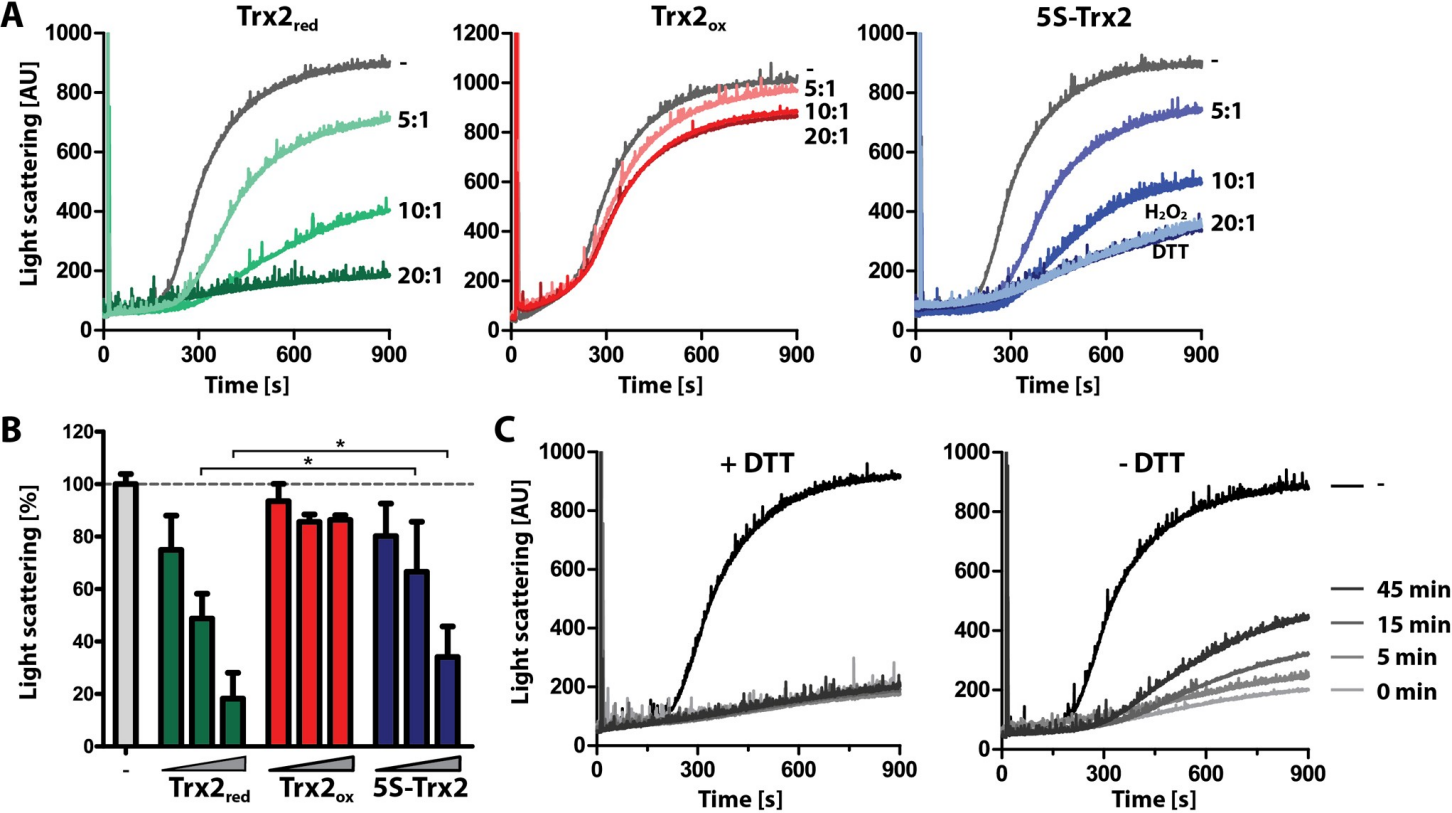
Reduced Trx2 and 5S-Trx2 prevent thermal aggregation of luciferase. (**A**) Native luciferase (0.1 μM) was incubated in the absence or presence of different molar ratios of either Trx2_red_, Trx2_ox_ or 5S-Trx2 at 44°C. Thermal aggregation of luciferase was followed by measuring light scattering at 360 nm. Trx2_red_ and Trx2_ox_ were prepared as described in Material and methods. For measuring the effect of Trx2_red_, the assay buffer was supplemented with 0.2 mM DTT which did not affect aggregation of luciferase in the absence of Trx2 or presence of 5S-Trx2. (**B**) Thermal aggregation of luciferase in the absence of Trx2 was set as 100% and the percentage of light scattering in the presence of a 5:1, 10:1 or 20:1 molar ratio of the different Trx2 species to luciferase was calculated from at least three independent assays. Student’s t-test (one-sample, unpaired) was used to evaluate significant differences between Trx2 and 5S-Trx2 (* = p < 0.05). (**C**) Trx2_red_ (20 μM) was pre-incubated in assay buffer at 44°C in the presence or absence of 2 mM DTT. After different times, the volume was increased ten-fold to the final assay volume, luciferase was added (0.1 μM), and light scattering was measured.

Based on the observation that Trx2, instead of stimulating the precipitation of the insoluble insulin B-chain, slowed down its aggregation (Fig 6), we wondered whether the protein might function as a molecular chaperone. To test this idea, we investigated the influence of Trx2 on the aggregation of thermally unfolding luciferase (Fig 7). Fully reduced (Trx2_red_) and oxidized (Trx2_ox_) protein were obtained as described in Materials and methods. DTNB assays revealed five moles of thiols per mole of Trx2_red_, and thus confirmed the complete reduction of the protein, and less than 0.8 thiols per Trx2_ox_ molecule.

Firefly luciferase rapidly unfolds and aggregates at temperatures above 42°C, a process that can be monitored by light scattering (Fig 7). In the presence of Trx2_red_, the aggregation was significantly decreased and almost completely suppressed at a 20:1 ratio of Trx2_red_ to luciferase (Fig 7A and 7B). In contrast, Trx2_ox_ only marginally affected luciferase aggregation even when used at a 20-fold excess. The cysteine-free 5S-Trx2, showed significant chaperone activity. The activity was slightly lower compared to Trx2_red_ but was unaffected by a treatment with DTT or H_2_O_2_ (Fig 7A).

To study how rapidly Trx2 loses its chaperone activity when exposed to non-reducing conditions, we incubated Trx2_red_ in the assay buffer at 44°C either in the presence or absence of DTT prior to measuring its chaperone activity (Fig 7C). While Trx2_red_ did not show any change in chaperone activity when kept under reducing conditions, absence of DTT in the assay buffer led to a gradual loss in chaperone activity with time. From these results, we concluded that Trx2 functions as a molecular chaperone, whose activity appears to be controlled by the oxidation status of its cysteine residues.

Next, we studied if Trx2 is also able to prevent the aggregation of chemically denatured proteins at 30°C. For these experiments, citrate synthase was treated with 6 M guanidine hydrochloride overnight. Light scattering was monitored upon dilution of denatured citrate synthase into HEPES buffer at 30°C. When the assays were conducted in the presence of Trx2_red_ or 5S-Trx2 that had been kept at 25°C, we did not observe any effect on the rate or degree of protein aggregation (S7 Fig). In contrast, when the two Trx2 variants were pre-heated for 5 min at 40°C before addition to the assay buffer, both Trx2_red_ and 5S-Trx2 slowed down the aggregation. These results suggested that elevated temperatures might activate the chaperone function of Trx2.

### Reduced monomeric Trx2 displays chaperone activity

**Fig 8:**
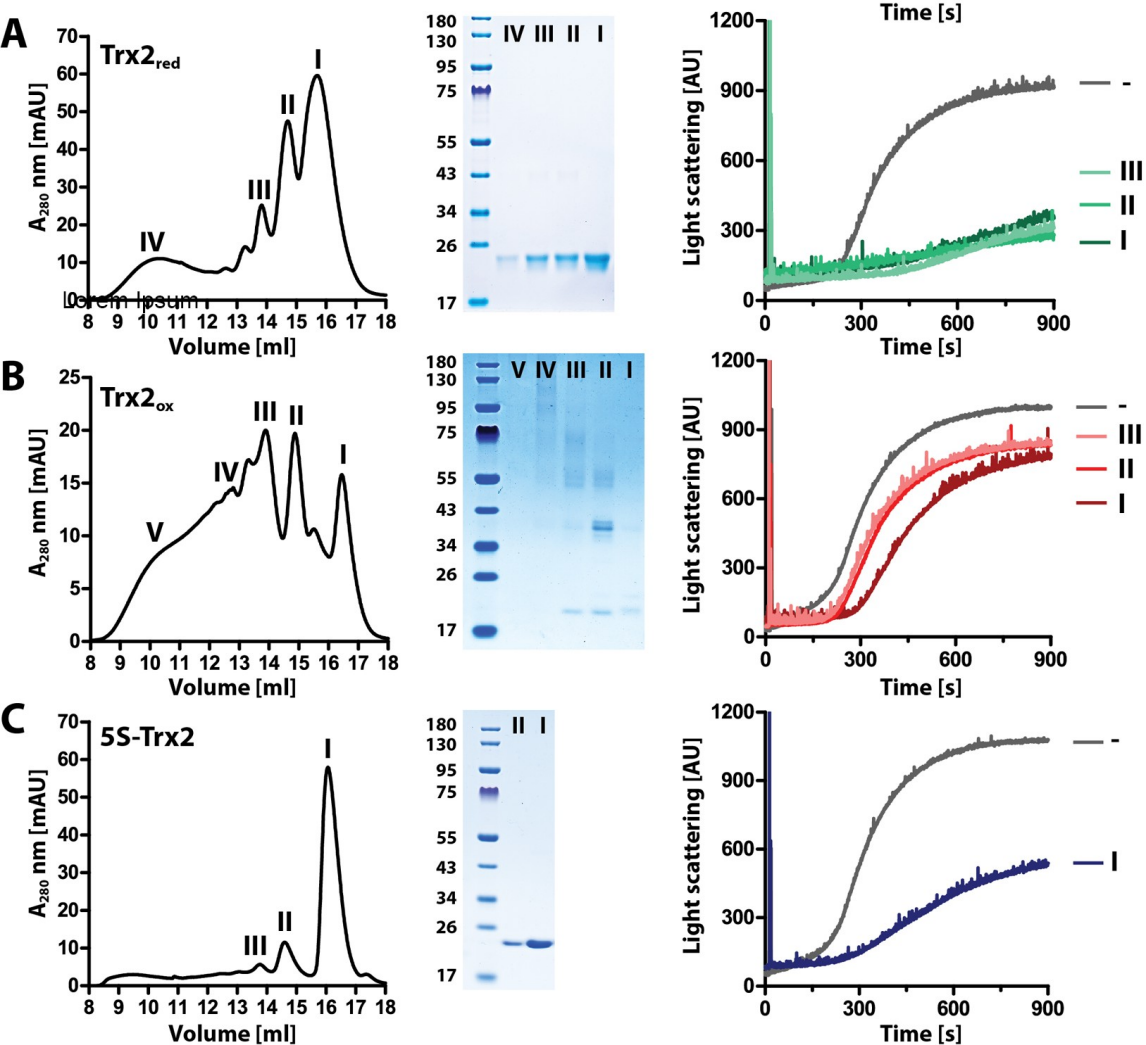
Monomeric, dimeric and oligomeric forms of reduced Trx2 have chaperone activity. Size exclusion chromatography on a Superdex 200 column of 200 μM of (**A**) Trx2_red_ in the presence of 1 mM DTT freshly added to the running buffer, (**B**) Trx2_ox_ and (**C**) 5S-Trx2 in the absence of DTT. Peak fractions (numbered) were collected and small aliquots were subjected to SDS-PAGE under non‐reducing conditions. The residual samples were used for studying the ability of the different Trx2 species to prevent thermal aggregation of native luciferase (0.1 μM) at 44°C. Trx2 was added at a 15:1 molar ratio to luciferase and light scattering was measured at 360 nm.

Analysis of WT-Trx2 by size exclusion chromatography revealed that the protein adopts a number of distinct forms (Fig 5). To investigate whether the redox and oligomerization states of the protein correlate with its chaperone function, we subjected Trx2_red_, Trx2_ox_ and 5S-Trx2 to gel filtration and analyzed the individual peak fractions by non-reducing SDS-PAGE and chaperone assays. As shown in Fig 8A and 8B, both Trx2_red_ and Trx2_ox_ eluted in several distinct peaks. SDS-PAGE of the four major peak fractions of Trx_red_ revealed the monomeric form, indicating that the protein is able to form non-covalent oligomers (Fig 8A). In contrast, SDS-PAGE of the peak fractions of Trx_ox_ showed an increasing number of covalently linked dimers, tetramers, and higher oligomeric species in the earlier fractions compared to primarily oxidized Trx2 monomers eluting in the later fractions (Fig 8B). These results confirmed the data shown in S6C Fig, and suggested that Trx2 forms intra- and intermolecular disulfide bonds upon oxidation. We then tested the major fractions of Trx_red_ for chaperone activity, and found them all to be similarly effective in preventing the thermal aggregation of luciferase at 44°C (Fig 8A). This result is not unexpected given that the different oligomeric states that we isolated from the size exclusion column will likely all adopt the same oligomeric state once diluted into the chaperone assay. None of the Trx2_ox_-containing fractions revealed any significant chaperone activity *in vitro* (Fig 8B). The 5S-Trx2 mutant eluted in a single prominent peak (Fig 8C), which appeared to correspond to peak 1 of Trx2_red_ and most likely represents the monomeric form of the protein (Fig 5). As before, the 5S-mutant showed chaperone activity similar to Trx2_red_ analyzed in the presence of DTT (Fig 8A and 8C). Together, these results suggest that Trx2 is chaperone-active in its monomeric conformation, and demonstrate that the formation of intra- and intermolecular disulfides abolishes the chaperone function.

### Reduced Trx2 and 5S-Trx2 support refolding of heat-inactivated luciferase

**Fig 9:**
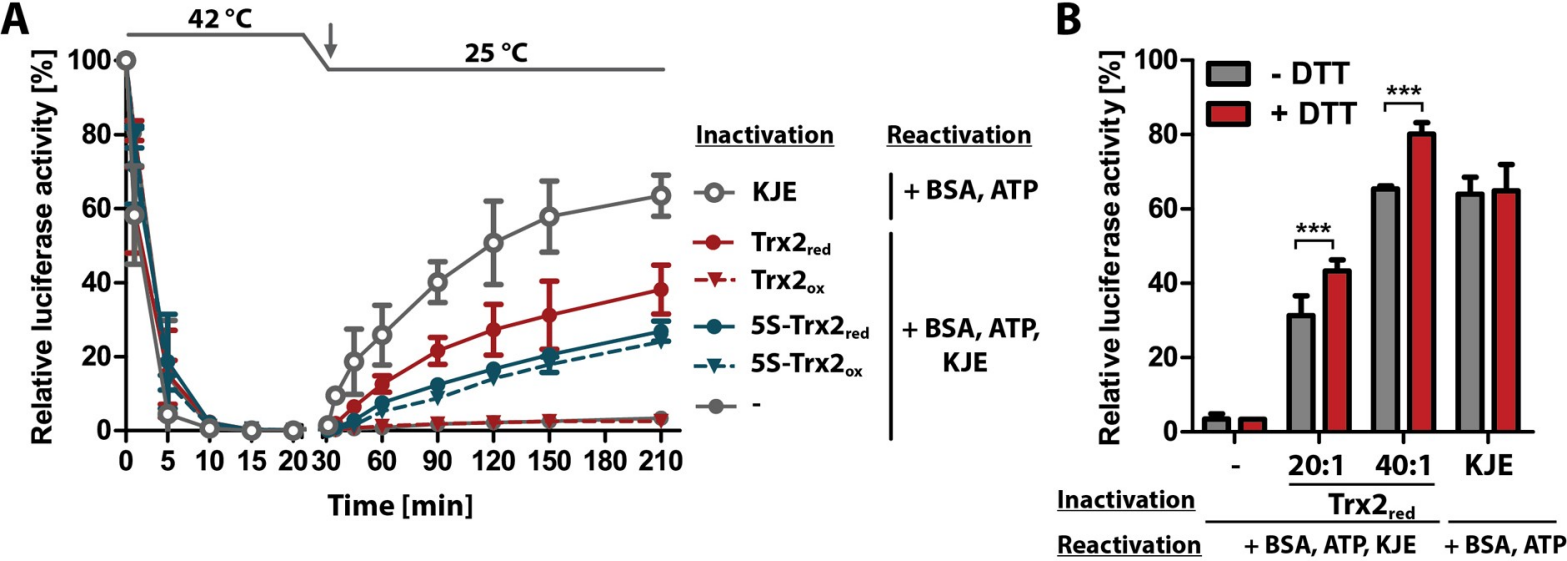
Reduced Trx2 and 5S-Trx2 maintain heat-inactivated luciferase in a refolding-competent state. (**A**) Luciferase (0.1 μM) was heat-inactivated for 20 min at 42°C in the absence or presence of a 20:1 molar ratio of Trx2 and 5S-Trx2 treated with DTT (red) or DTT followed by H_2_O_2_ (ox) as described in Material and methods. Samples were reset to 25°C and after 10 min, mixed with 0.1 mg/ml BSA, 2 mM MgATP, and a 20:4:20:1 ratio of DnaK:DnaJ:GrpE (KJE) system to luciferase (arrow). As a control, luciferase was heat‐inactivated in the presence of a 20:4:20:1 ratio of the KJE system and after cooling, 2 mM MgATP and 0.1 mg/ml BSA were added (arrow). At different time points, aliquots were collected and luciferase activity was measured. (**B**) Luciferase (0.1 μM) was inactivated at 42°C in the presence of a 20:1 or 40:1 molar ratio of Trx2_red_ or a 20:4:20:1 ratio of the KJE system to luciferase either in the absence or presence of 0.2 mM DTT for 20 min. After cooling the samples to 25°C for 10 min, 0.1 mg/ml BSA and 2 mM MgATP were added. All samples except those containing already the KJE system were supplemented with a 20:4:20:1 ratio of KJE to luciferase. Luciferase activity was measured after 180 min of incubation at 25°C. The luciferase activity measured before the inactivation at 42°C was set to 100%. Student’s t-test (one-sample, unpaired) was used to evaluate significant differences between samples with and without DTT (*** = p < 0.001).

Our studies showed that Trx2_red_ and 5S-Trx2 prevent the thermal aggregation of luciferase *in vitro* whereas Trx2_ox_ had no significant effect. To further examine whether the chaperone-active conformations of Trx2 affect the rate of thermal inactivation and/or the refolding of luciferase upon return to non-stress temperatures, we incubated luciferase at 42°C in the absence or presence of Trx2_red_, Trx2_ox_ or 5S-Trx2. At this temperature, luciferase activity decreased within 20 min to less than 1% of its initial activity (S8 Fig). After resetting the temperature to 25°C and adding ATP, we did not observe any significant luciferase reactivation in the absence or presence of Trx2. Since many ATP-independent chaperones are unable to refold their client proteins but can maintain them in a refolding competent state and hand them over to ATP-dependent foldases, we supplemented the reaction mixture after its shift to 25°C with the DnaK/DnaJ/GrpE (KJE) foldase system. This chaperone system is highly homologous to the mitochondrial Hsp70 system [48]. It is known to promote reactivation of unfolding protein intermediates that were kept refolding-competent by chaperone holdases, such as Hsp33 or the small Hsps, but is unable to refold fully aggregated proteins [49]. Upon addition of the KJE system, we observed substantial refolding of luciferase that was heat-inactivated in the presence of Trx2_red_ or 5S-Trx2 but not of luciferase that was heat-inactivated in the absence of Trx2_red_ or in the presence of Trx2_ox_ (Fig 9A). Since Trx2_red_ was highly sensitive to spontaneous oxidation (Figs 5 and 7C and S6 Fig), we also performed the luciferase heat-inactivation and refolding with different amounts of Trx2_red_ in the absence or presence of 0.2 mM DTT (Fig 9B). Reactivation of luciferase, inactivated in the absence of any chaperone or in the presence of the KJE-system, was not affected by DTT. In contrast, the ability of Trx2 to maintain luciferase in a folding competent state was significantly enhanced when the assay was performed in the presence of DTT. These results strongly suggested that chaperone-active Trx2_red_ and 5S-Trx2 are able to maintain thermally unfolding luciferase in a refolding-competent conformation, and can transfer the client protein to the KJE-system for refolding once non-denaturing conditions are restored.

### Reduced Trx2 and 5S-Trx2 increase surface hydrophobicity at heat-shock temperatures

**Fig 10:**
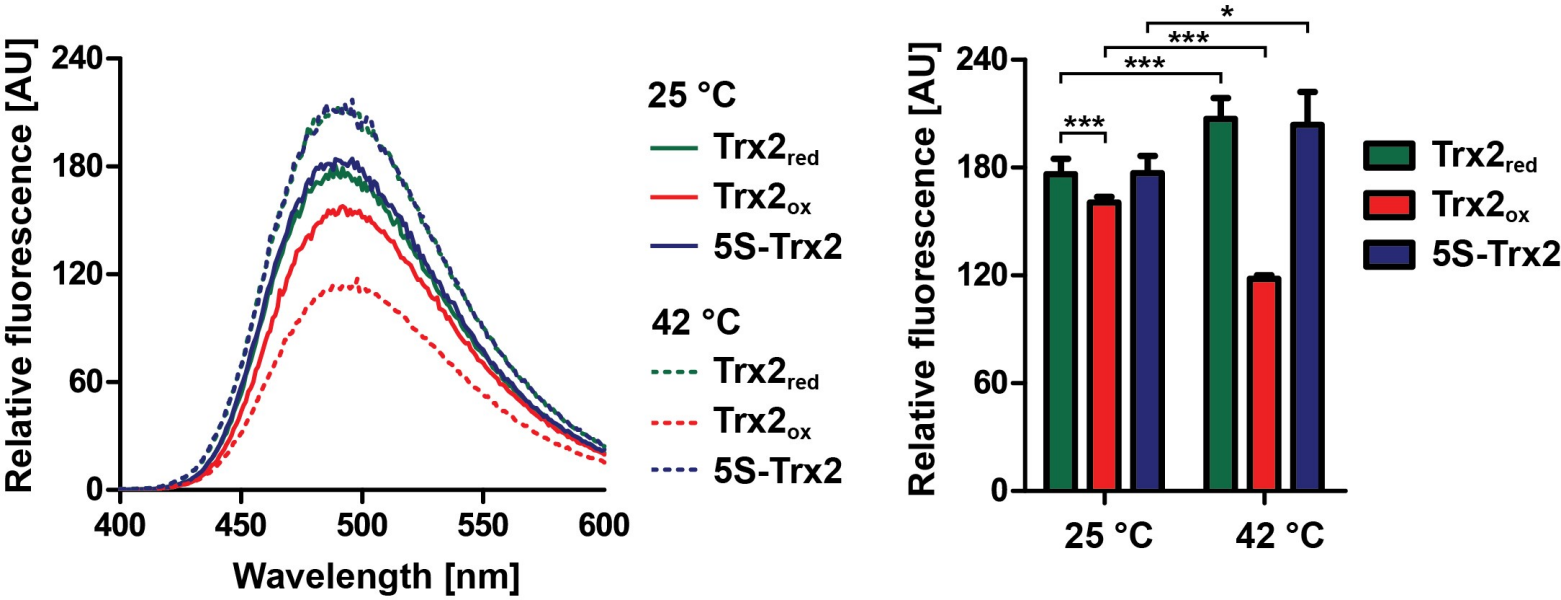
Temperature-dependent structural changes of Trx2. Surface hydrophobicity of Trx2_red_, Trx2_ox_ and 5S-Trx2 at 25°C (solid lines) or 42°C (dashed lines) was analyzed by Bis-ANS binding and fluorescence spectroscopy. For each protein, the peak value was determined for at least three independent measurements. Student’s t-test (one-sample, unpaired) was used to evaluate significant differences between samples (* = p < 0.05 and *** = p < 0.001).

As described above, Trx2_red_ and 5S-Trx2 prevent aggregation of unfolding proteins when incubated at elevated temperatures (Fig 7 and S7 Fig). To test whether elevated temperatures result in structural changes that might be required for the activation of the chaperone function, we compared secondary structure elements in Trx2_ox_, Trx2_red_ and 5S-Trx2 by measuring far-UV circular dichroism (CD) spectra and surface hydrophobicity via 4,4’-bis-anilino-1,1’-binaphthyl-5,5’-dissulfonic acid (bis-ANS) binding at both 25°C and 42°C. We did not detect any significant differences in the secondary structure arrangement of the proteins at either temperature, and no major difference in the temperature transition (S9 Fig). However, the bis-ANS fluorescence signal of both Trx2_red_ and 5S-Trx2 samples showed a significant increase when the temperature was raised from 25°C to 42°C (Fig 10) indicating that the proteins expose hydrophobic patches as the temperature increases. This finding was entirely consistent with the behavior of other temperature-activated chaperones [50–52]. In contrast, the chaperone-inactive Trx2_ox_ showed a much lower bis-ANS fluorescence compared to Trx2_red_ and 5S-Trx2 at 25°C and an even further reduction of the signal upon incubation at 42°C. These results suggest that oxidative disulfide bond formation causes pronounced structural rearrangements in Trx2 that reduce surface hydrophobicity and may explain why Trx2_ox_ is unable to interact with unfolding client proteins.

## Discussion

Here we show that *T*. *brucei* Trx2, a Trx-type protein that lacks any counterpart outside the order Kinetoplastida, is important for proliferation, cell survival upon prolonged heat stress and infectivity of the parasites. Trx2 is a mitochondrial protein. Indeed, ablation of archaic translocase of the mitochondrial outer membrane (ATOM 40), which is essential for the import of mitochondrial precursor proteins into the mitochondrion of *T*. *brucei*, strongly reduces the abundance of Trx2 in the mitochondrion [23]. Cell lysates often revealed two Trx2 bands which most likely are both processed forms with the upper one representing an intermediate. *T*. *brucei* possesses a canonical mitochondrial processing peptidase (MPP) as well as a mitochondrial intermediate peptidase (corresponding to Oct1 in yeast) [25, 53] which appears to act synergistically with MPP [53]. Alternatively, the pre-sequence may be removed by two consecutive cleavage steps both performed by MPP as shown for yeast and human frataxin [54]. The 42 N-terminal residues of Trx2 comprise ten arginine residues of which several may serve as cleavage sites of MPP [24].

Interestingly, silencing of Mic20, a Trx-like subunit of the mitochondrial contact site and cristae organization system (MICOS) complex in the mitochondrial IMS, induces the upregulation of Trx2 in PC *T*. *brucei* together with several chaperone-like proteins [55]. A role of Trx2 in cristae formation is unlikely as the mitochondrion of BS cells is devoid of cristae. However, Mic20 appears to be involved also in the import of proteins into the IMS and matrix of the mitochondrion [55]. A putative role of Trx2 in folding/stabilization of proteins that are imported into the mitochondrion would be in accordance with its chaperone-like activities *in vitro*.

Remarkably, a cysteine-free mutant of Trx2 was able to fully substitute for the authentic protein. The finding that cells in which Trx2 was down-regulated displayed the same sensitivity towards paraquat as WT parasites and WT-Trx2 or 5S-Trx2 cKO cells, indicates that Trx2 does not play a role in the oxidative stress response of the parasite. Depletion of Trx2 by RNAi attenuated the infectivity and viability of *T*. *brucei* in the mouse model. Importantly, 5S-Trx2 cKO parasites were at least as infectious as WT-Trx2 cKO cells. This clearly showed that the physiological role of Trx2 is thiol-independent.

The absorption spectra of dimeric and oligomeric forms of recombinant Trx2 suggested that the protein can coordinate iron sulfur clusters. Only a small subset of Trx-fold proteins, most of them representing mono- or dithiol glutaredoxins [13, 26, 43, 56, 57], have been shown to bind iron sulfur clusters. The first natural Trx found to bind an iron sulfur cluster is IsTRP, a protein from the tapeworm *Echinococcus granulosus* [44]. However, the primary structures of IsTRP and *T*. *brucei* Trx2 do not display any pronounced similarity. The fact that the 5S-mutant could replace the authentic Trx2 *in vitro* and *in vivo* indicates that iron sulfur cluster binding is not an essential physiological role of *T*. *brucei* Trx2.

Recombinant *T*. *brucei* Trx2 lacked protein disulfide reductase activity but, instead, slowed down precipitation of the insoluble B-chain in the insulin reduction assay. *AtTDX*, a Trx-like protein from *Arabidopsis thaliana*, has been reported to have insulin reductase activity at low concentrations which is lost when higher concentrations are applied [58]. This is not the case for Trx2 as the parasite protein did not display reductase activity at any of the concentrations tested. Trx2 species that lacked free cysteine residues were even more efficient in preventing precipitation suggesting that the protein can function as a thiol-independent molecular chaperone. Indeed, Trx2_red_ and the 5S-Trx2 mutant, but not Trx2_ox_, slowed down the aggregation of heat-denatured luciferase and were able to maintain a thermally unfolding protein in a conformation that allows transfer to the DnaK/DnaJ/GrpE system for refolding. When pre-heated at 40°C, Trx2_red_ and 5S-Trx2 prevented also the aggregation of chemically denatured citrate synthase indicating that elevated temperatures trigger the chaperone function.

Trx2_red_ and Trx2_ox_ eluted from gel filtration columns as several distinct oligomeric forms. None of the Trx_ox_ species was chaperone-active. In contrast, all Trx2_red_ species were composed of reduced monomers and displayed chaperone activity. In *AtTDX*, the holdase activity is associated with the formation of oligomeric forms of the protein [58]. However, oligomerization does not appear to be a general mechanism for Trx-type proteins to act as chaperones. The expression of recombinant proteins as fusion proteins with Trx, to increase their solubility and favor folding, supports our conclusion that Trx2_red_ is chaperone-active in monomeric form. Interestingly, 5S-Trx2 and the monomeric form of Trx2_red_ displayed an apparent mass that was nearly twice that of the calculated protein mass. In addition, the surface hydrophobicity of both species proved to be significantly higher than that of Trx2_ox_ and further increased when the temperature was raised from 25°C to 42°C. This indicates that Trx2_red_ and 5S-Trx2 adopt a conformation that supports binding of client proteins which becomes even more pronounced at higher temperature and may explain why Trx2_ox_, for which the surface hydrophobicity was low and further dropped at elevated temperatures, lacks chaperone activity. Thiol-independent chaperone functions have been shown for *E*. *coli* Trx [59] and various other Trx-type proteins [60, 61] whereby most of these proteins display both reductase and chaperone activity.

The properties of *T*. *brucei* Trx2 revealed in this work are remarkably reminiscent of those reported for the mitochondrial 2-Cys-peroxiredoxin (mPrx, mTXNPx) from *Leishmania infantum* [51, 62, 63]. Both proteins are located in the single mitochondrion of the respective parasites, confer heat tolerance to the cells and are critical for infectivity. Recombinant Trx2 and mPrx have a high propensity to air-oxidize and act as thiol-independent chaperones for putative client proteins under reducing, but not under non-reducing conditions. Both proteins cooperate with the bacterial DnaK/DnaJ/GrpE (KJE) system, which is highly homologous to the mitochondrial Hsp70 system [51], and their chaperone-active reduced forms maintain client proteins in a refolding-competent conformation. An *in silico* survey of the Hsp70/J-protein machinery of African trypanosomes revealed 12 putative Hsp70 proteins and 67 putative J-proteins [64]. Many of these proteins were predicted or experimentally shown to be localized in the mitochondrion. It will be interesting to see which of these Hsp70/J-protein couples may interact with Trx2. In a first attempt to identify proteins that may interact with Trx2, we subjected WT-Trx2, C63/66S-Trx2 and 5S-Trx2 cKO cells to co-immunoprecipitation and label-free quantitative mass spectrometry (S10 Fig and S1 Text). This approach did not yield a specific interaction partner.

Many stress-activated chaperones serve as dual-function proteins with distinct and mutually exclusive activities under non-stress conditions [65]. For the parasite mPrx and Trx2, an essential redox activity can be ruled out as both proteins can be replaced by cysteine-free mutants. The mPrx is required for long term stability of the insect stage of *L*. *infantum* at 37°C but is dispensable when the parasites are cultured at 25°C [62]. In contrast, the presence of Trx2 or 5S-Trx2 was required for proliferation of PC *T*. *brucei* under standard culture conditions at 27°C and thus in the absence of a known stress. *T*. *brucei* mPrx shares 70% of all residues with the *Leishmania* protein suggesting largely conserved function(s). RNAi against mPrx in BS *T*. *brucei* does not cause any proliferation defect [8]. Our finding that Trx2 is required in both BS and PC *T*. *brucei* indicates that the mPrx is unable to functionally substitute for Trx2. Trx2 and mPrx may act as chaperones on distinct client proteins and/or Trx2 plays an additional constitutive physiological role. Such a dual function has been shown for the mitochondrial Hsp70. A small fraction of the protein is involved in pre-protein import whereas the majority of mitochondrial Hsp70 is dedicated to protein folding in the mitochondrial matrix [66, 67]. As shown recently, heat-stress does not induce a general mitochondrial protein aggregation but, remarkably, decreases the import efficiency for cytosolic proteins [68]. If the proliferation defect of Trx2-depleted cells observed at 27°C is indeed due to an impaired mitochondrial protein import one may expect that this process is even more affected when the cells were exposed to 37°C. Future work should focus on the specific role of the novel molecular chaperone in the mitochondrion of African trypanosomes.

## Materials and methods

### Materials

Puromycin dihydrochloride, hygromycin B and blasticidin were purchased from Roth, Karlsruhe, Germany. DAPI, tetracycline (Tet), insulin, paraquat and 2-mercaptoethanol were from Sigma-Aldrich. Fetal calf serum (FCS) was from Biochrome. H_2_O_2_ was from Merck. All restriction enzymes were purchased from ThermoFisher. Primers were synthesized by Eurofins MWG Operons, Ebersberg, Germany. Plasmids were sequenced by GATC Biotech AG, Konstanz, Germany. The pET vectors were a gift of Gunter Stier, BZH, Heidelberg University. The pHD vectors as well as rabbit antibodies against *T*. *brucei* aldolase were kindly provided by Dr. Christine Clayton, ZMBH, Heidelberg University. The pRPa vector was obtained from Dr. Keith Matthews, Edinburgh. Antibodies against *T*. *brucei* cytochrome c (Cytc) were provided by Dr. André Schneider, Bern. Recombinant *T*. *brucei* Tpx [69], rabbit antibodies against *T*. *brucei* lipoamide dehydrogenase [27] and guinea pig antibodies against mitochondrial peroxiredoxin (mPrx; TriTrypDB: Tb927.8.1990) [12] were obtained previously. Guinea pig antibodies against *T*. *brucei* Trx2 (TriTrypDB: Tb427.03.4240) were generated by Eurogentec, Seraing, Belgium. Mouse anti-c-myc and HRP-conjugated goat antibodies against mouse IgGs were from Santa Cruz Biotechnology. HRP-conjugated goat antibodies against rabbit IgGs were purchased from ThermoFisher. HRP-conjugated donkey antibodies against guinea pig IgGs were from Merck.

### *Trypanosoma brucei* cell lines and culture

Culture-adapted BS and PC *Trypanosoma brucei* of cell line 449 were used. The cells are descendants of strain Lister 427 that were stably transfected with pHD449 encoding the tetracycline repressor [70]. 2T1 cells derived from BS 449 cells, which contain a 3’-*HYG* fragment and *VSG* expression site promoter-driven *PAC* ORF [71], were used to generate BS cell lines overexpressing Trx2-myc_6_. Unless otherwise stated, BS cells were grown in HMI-9 medium at 37°C in a humidified atmosphere with 5% CO_2_, and PC cells were cultivated at 27°C in MEM-Pros medium, both supplemented with 50 U/ml penicillin, 50 mg/ml streptomycin and 10% FCS as described previously [35]. HMI-9 and MEM-Pros media contained 0.2 μg/ml and 0.5 μg/ml phleomycin, respectively. BS and PC 449 cells were used to generate Trx2 RNAi and cKO cell lines. For phenotypic analyses, cells were grown in the presence or absence of 1 μg/ml Tet without selecting antibiotics.

### Cloning of constructs for overexpression, RNA interference, deletion, and mutagenesis of Trx2 in *T*. *brucei*

Genomic DNA was isolated from BS *T*. *brucei* parasites using the Qiagen DNeasy Blood and Tissue kit. Unless otherwise stated, *E*. *coli* NovaBlue competent cells (Merck) were used for plasmid amplification. All primer sequences are listed in S1 Table. For Tet-inducible overexpression of Trx2-myc_6_ in BS 2T1 cells, the coding region of *trx2* was amplified by PCR using Trx2-myc_6_-*Hind*III-F and Trx2-myc_6_-*Xba*I-R as primers and genomic DNA as template and cloned into the pRPa vector [71] yielding the pRPa-Trx2-myc_6_ plasmid. For Tet-inducible expression of Trx2 in PC cells, the coding region of *trx2* was amplified using Trx2-myc_2_-*Hind*III-F and Trx2-myc_2_-*BamH*I-R primers and cloned into the pHD1700 vector yielding the pHD1700-Trx2-myc_2_ plasmid.

For Tet-inducible depletion of Trx2 by RNAi, a stem loop construct targeting the coding region of *trx2* was assembled. The fragment was selected using the RNAit primer design algorithm to minimize off-target effects [72]. With the primer pairs Trx2i-*HpaI*/Trx2i-*EcoRI*-1 or Trx2i-*HindIII*/Trx2i-*EcoRI*-2 and genomic DNA as template, a 345 and 300 bp DNA fragment, respectively, was amplified by PCR. Both fragments were digested with *EcoR*I, ligated and cloned into the pGEM-T easy vector (Promega). Following *Hind*III/*Hpa*I digestion, the cassette was cloned into the pHD678 vector (hygromycin resistance) [70]. For both cloning steps to generate the pHD678-Trx2i plasmid, SURE *E*. *coli* competent cells (Stratagene, Aglient Technologies) were used.

To replace both *trx2* alleles, the 5’untranslated region (UTR) was amplified by PCR using the primers Trx2-5’UTR-*Xho*I and Trx2-5’UTR-*Hind*III yielding a fragment of 237 bp. The 3’UTR of *trx2* was amplified using Trx2-3’UTR-*Pst*I and Trx2-3’UTR-*Not*I generating a fragment of 287 bp. Both fragments were purified and cloned into the pHD1747 (puromycin resistance) and pHD1748 (blasticidin resistance) vectors to generate the pHD1747-Trx2-KO and pHD1748-Trx2-KO plasmids.

For mutageneses, the QuickChange II site-directed mutagenesis Kit (Agilent Technologies) was used. With pHD1700-Trx2-myc_2_ as template and *PfuUltra* HF DNA polymerase, the cysteines in Trx2 were replaced by serine residues. The first construct generated was pHD1700-C63/66S-Trx2-myc_2_ which then served as template for the sequential replacement of the other cysteine residues yielding finally pHD1700-5S-Trx2-myc_2_. The amplicons obtained were digested with *Dpn*I and used to transform competent *E*. *coli* cells. The mutations were verified by plasmid sequencing.

### Generation of Trx2 overexpressing, RNAi and cKO *T*. *brucei* cell lines

For all transfections, approximately 4 x 10^7^ cells were harvested and 10 μg of digested and ethanol precipitated plasmid DNA was used. Transfections were carried out in the Amaxa Nucleofector electroporator with program X-001 using either the Amaxa transfection solution (Lonza) or a buffer developed for the transfection of BS *T*. *brucei* [73]. For the generation of BS 2T1 cells overexpressing Trx2-myc_6_, the pRPa-Trx2-myc_6_ was linearized with *Asc*I. Stably transfected cell lines were selected with 2.5 μg/ml hygromycin. To produce Tet-inducible Trx2 RNAi cell lines, WT *T*. *brucei* were transfected with the *NotI*-linearised pHD678-Trx2i plasmid. Stably transfected BS and PC clones were selected with 10 μg/ml and 150 μg/ml hygromycin, respectively.

To generate Trx2 cKO cells, BS and PC WT *T*. *brucei* were firstly transfected with *NotI*/*XhoI*-digested pHD1747-Trx2-KO and selected with 0.2 μg/ml and 2 μg/ml puromycin, respectively. The single KO cells obtained were subsequently transfected with the *NotI* linearized pHD1700-Trx2-myc_2_, pHD1700-C63/66S-Trx2-myc_2_ or pHD1700-5S-Trx2-myc_2_ plasmid DNA and cultured in the presence of puromycin plus 10 μg/ml or 150 μg/ml hygromycin to select for BS and PC cell lines, as well as 1 μg/ml Tet. To replace the second *trx2* allele, these clones were transfected with the *NotI*/*XhoI*-digested pHD1748-Trx2-KO construct, selecting BS and PC clones with puromycin, hygromycin, Tet, as well as 5 μg/ml and 10 μg/ml blasticidin, respectively. Replacement of both *trx2* alleles by the resistance genes was confirmed by PCR analyses. BS and PC RNAi and cKO cells were continuously cultured in the presence of 10 and 50 μg/ml hygromycin, respectively.

### Cultivation of PC *T*. *brucei* under stress conditions

In all experiments, the starting density was 5 x 10^5^ cells/ml. The cKO cells were maintained in the presence of 1 μg/ml Tet unless otherwise stated. To induce a putative endoplasmic reticulum stress, cells were incubated with different concentrations of DTT. To induce oxidative stress, cells were treated with different concentrations of H_2_O_2_ or paraquat. Either short term cell viability or proliferation was followed. For heat shock induction, MEM-Pros medium was pre-heated to 41°C for at least 3 h before adding the cells. For long term heat stress, 5S-Trx2 cKO cells were firstly maintained at 27°C in the presence or absence of Tet. When cKO cells grown in the absence of Tet stopped proliferating (usually observed after 6 to 9 days -Tet), they were transferred to 37°C. Every 24 h, the cells were counted and diluted to the start density.

### Fractionated digitonin lysis of PC *T*. *brucei*

The differential membrane permeabilization was done essentially as described previously [26], except that the 10 mM Tris-HCl, 150 mM NaCl, 1 mM EDTA, pH 8.0 buffer was supplemented with 0.1 mM PMSF, 150 nM pepstatin and 4 nM cystatin and the lysis was done for 4 min on ice. On two gels, the equivalents of 1 x 10^7^ cells were applied per lane and the blots developed with antibodies against Trx2 followed by Cytc antibodies. For the next couple of gels, 2 x 10^7^ cells and antibodies against Grx2 and C-Myc were used. For the third two gels, 2 x 10^6^ cells and antibodies against mPrx and Tpx were applied. For the subsequent Western blot analyses, the antibodies against Trx2, c-Myc, mPrx, Grx2, Cytc and Tpx were diluted 1:1,000, 1:400, 1:2,000, 1:1,000, 1:200 and 1:2,000, respectively. In each case, the blots were developed with the second antibody after reactivation without stripping. For the secondary antibodies and further details see next section.

### Western blot analysis

Cells were harvested, washed, resuspended in PBS containing 20 mM DTT, incubated for 30 min at 30°C, and mixed with 4 x SDS sample buffer containing 8 M urea or directly mixed with reducing sample buffer and incubated for 30 min at 30°C. Total lysates from 1–3 x 10^7^ cells were separated on 12 or 14% SDS gels. After electrophoresis, proteins were transferred onto a 0.2 μm PVDF membrane (GE Healthcare) and probed with the *T*. *brucei* Trx2 antibodies (1:1,000) overnight followed by HRP-conjugated donkey antibodies against guinea pig IgGs (1:40,000). For a loading control, membranes were treated with antibodies against *T*. *brucei* aldolase (1:20,000) or lipoamide dehydrogenase (LipDH) (1:20,000) followed by HRP-conjugated goat antibodies against rabbit IgGs (1:20,000). For detection of the ectopically expressed myc-tagged Trx2 species, mouse anti-c-myc antibody (1:400) followed by HRP-conjugated goat antibodies against mouse IgG (1:20,000) were used. Bands were visualized by chemiluminesence using the SuperSignal West Pico or Femto substrate (ThermoFisher) or the Western BLoT Ultra Substrate (Takara) and a digital imager (GE Healthcare).

### Determination of the cellular concentration and mass of mature Trx2 in PC *T*. *brucei*

To estimate the cellular level of Trx2, between 0.5 and 5 ng recombinant Trx2 and the lysate of 1 to 3 x 10^7^ PC *T*. *brucei* were separated on 14% SDS gels, blotted and probed with the *T*. *brucei* Trx2 antibodies as described for Western blot analysis. ImageJ was used to quantify the raw integrated density (the sum of the values of the pixels) of each band. The density of the band corresponding to the highest amount of recombinant Trx2 was set as 100% and the % signal for other protein bands was calculated as a proportion of this. The signals from the two forms of Trx2 detected in the cell lysates were combined to give the total cellular Trx2. The values from seven independent analyses were averaged. To determine the size of the two Trx2 species detected in the cell lysates, the relative mobility of both bands and of recombinant Trx2 was measured and the molecular mass calculated based on standard curves derived from PageRuler Plus (ThermoFisher) and Precision Plus Protein DualColor (BioRad) protein standards.

### Immunofluorescence microscopy

Immunofluorescence microscopy was conducted as described previously [7]. Approximately 2 x 10^6^ BS and PC cells inducibly expressing myc-tagged versions of Trx2 grown in the presence of Tet were harvested, washed with PBS, and stained with MitoTracker Red CMXRos (Life Technologies). Afterwards, the cells were fixed, permeabilized and treated with anti-c-myc antibodies (1:200 in 0.5% gelatin in PBS) for 1 h at room temperature followed by goat anti-mouse antibodies coupled to Alexa Fluor 488 (1:1,000 in 0.5% gelatin in PBS, Molecular Probes). The nucleus and kinetoplast were visualized by DAPI staining. Cells were examined under a Carl Zeiss Axiovert 200 M microscope equipped with an AxioCam MRm digital camera using the AxioVision program (Zeiss, Jena).

### Ethics statement

The animal experimentation protocol used in this work was approved by the Animal Use and Ethic Committee (CEUA) of the Institut Pasteur de Montevideo (Protocol 001–18). It is in accordance with the Federation of European Laboratory Animal Experimentation (FELASA) guidelines and the National Law for Laboratory Animal Experimentation (Law nr. 18.611).

### Mouse infection with *T*. *brucei*

The infection experiments were carried out using Balb/cJ female mice (7–9 weeks old) hosted at the Transgenic and Experimental Animal Unit (Institut Pasteur de Montevideo) as described previously [27]. Half of the animals was fed with water containing 1 mg/ml oxytetracycline 96 h prior to infection and during the course of the experiment, replenishing the water with fresh drug every 48 h. Mice (five per group) fed with plain water or oxytetracycline (+Tet groups) were infected intraperitoneally with 10^4^ exponentially growing WT BS *T*. *brucei*, Trx2 RNAi, WT-Trx2 cKO or 5S-Trx2 cKO cell lines. The health status and survival of the animals were monitored daily. The parasitemia levels were assessed from day 3 post-infection onwards. Briefly, blood taken from the submandibular sinus (1–100 μl) was collected in a tube containing 5 μl of anticoagulant (equilibrated solution of sodium and potassium EDTA salts at 0.342 mol/l, pH 7.2; anticoagulant W, Wiener lab). For some samples, 10 μl PBS-1% (w/v) glucose was added to the blood to extent parasite viability during sample processing. After thorough homogenization, an aliquot was diluted 1:20 in red cell lysis buffer (0.8% (w/v) NH_4_Cl, 0.084% (w/v) NaHCO_3_ and 0.038% (w/v) Na_2_-EDTA, pH 7.4), incubated for 2 min at room temperature, and further diluted with PBS-1% (w/v) glucose when the parasite density was above 10^6^ cells/ml. Parasites were counted under an inverted microscope using a Neubauer chamber, which allows to detect a minimum parasite density corresponding to 2.5 × 10^4^ cells/ml. Mice showing an impaired health status and/or a parasite load of ≥ 10^8^ cells/ml blood were euthanized. The Kruskal-Wallis test (followed by Dunn's multiple comparison) and/or the Mann Whitney test (non-parametric, two-tailed) were applied to assess statistical significance of parasitemia. Survival plots were analyzed using the *log rank test*. The statistical analysis was performed with GraphPad Prism version 6.01 for Windows (GraphPad Software, La Jolla, California, USA). P values < 0.05 were considered statistically significant.

### Cloning, expression, and purification of recombinant Trx2 species

The *trx2* coding region without putative mitochondrial pre-sequence was amplified by PCR from genomic DNA using the primer pair Trx2 Short *NcoI*-F/Trx2 *Acc*65I-R. The amplicon was purified and cloned into the pET-MBP-vector. To generate Trx2 species in which either Cys63 and Cys66 or all five cysteines were replaced by serine residues, the pET-MBP-Trx2-short plasmid was subjected to site-directed mutagenesis as outlined above for pHD1700-Trx2. The full length coding region (Trx2 fl) was amplified using the primer couple Long *NcoI*-F/Trx2 *Acc*65I-R and cloned into the pET-NusA-Trx2 vector. Competent BL21 (DE3) *E*. *coli* cells were transformed with the respective pET-Trx2 plasmid. Three liters of bacterial cell culture were grown in LB medium and overexpression of the different *T*. *brucei* Trx2 species induced with 0.1 mM IPTG. After overnight cultivation at 18°C, the cells were harvested and suspended in 50 ml buffer A (50 mM sodium phosphate, 300 mM NaCl, pH 8.0) containing 50 μM PMSF, 150 nM pepstatin, 4 nM cystatin, 5 mg lysozyme and 0.5 mg DNase. The cells were disintegrated by sonication and the cell debris removed by centrifugation. The recombinant proteins were purified by three consecutive chromatographies on Ni-NTA-Superflow columns (Qiagen) using buffer A with different imidazole concentrations. The supernatant was loaded on a 12 ml Ni-NTA-column equilibrated with buffer A and connected to an ÄKTA Pure system (GE Healthcare). The column was washed with 25 mM imidazole. The fusion protein was eluted with 150 mM imidazole, re-buffered to buffer A, and concentrated using an Amicon Ultra 30 kDa cut-off concentrator (Millipore). In a total volume of 5 ml, the fusion protein was treated for 1 h at room temperature followed by 16 h at 4°C with 2 mg of His-tagged TEV-protease [74]. The digest was applied onto a 5 ml Ni-NTA column equilibrated in buffer A. The tag-free Trx2 was eluted with 25 mM imidazole, washed with buffer A for buffer exchange and concentrated in an Amicon Ultra 10 kDa cut-off concentrator. To remove any remaining fusion protein or impurity, the last purification step was repeated, Trx2 was eluted with 10 to 25 mM imidazole and treated as before. The concentration of the tag-free Trx2 species was determined by Bradford assay and the pure proteins stored at 4°C in the presence of 0.02% sodium azide.

### Determination of free thiol groups

The concentration of free SH groups was determined by reaction with 5,5’-dithiobis-(2-nitrobenzoic acid (DTNB, Ellman’s reagent, ε_412_ = 13.6 mM^-1^cm^-1^).

### Generation of reduced and oxidized forms of Trx2

Recombinant Trx2 and 5S-Trx2 (200 μM) were treated with 5 mM DTT in 40 mM HEPES-KOH, pH 7.5 for 30 min at 30°C and washed with buffer on an Amicon filter with a 10 kDa cut-off until the flow-through was free of thiols (measured by adding DTNB). To generate oxidized Trx2 (Trx2_ox_), the reduced Trx2 (Trx2_red_) was again diluted to 200 μM in buffer and incubated with 2 mM H_2_O_2_ for 30 min at 30°C. H_2_O_2_ was removed and the protein concentrated as described above. The 5S-Trx2 variant was also treated with DTT and subsequently H_2_O_2_ to exclude any non-thiol-based oxidative modifications.

### UV-visible and CD-spectroscopy

The absorption spectra of recombinant Trx2, C63/66S-Trx2 and 5S-Trx2 were recorded on a Jasco 650 spectrophotometer. Trx2_red_, Trx2_ox_ and 5S-Trx2 were diluted to 0.2 mg/ml in 20 mM KH_2_PO_4_, pH 7.5 (filtered and degassed) and far-UV CD spectra recorded between 260–190 nm in a quartz cell with 1 mm path-length at 25°C and 42°C using a Jasco-J810 spectropolarimeter. To monitor the thermostability of Trx2, the CD signal at 222 nm was followed from 20°C to 80°C. The temperature was controlled by a Jasco Peltier device and increased at a rate of 1°C/min. All spectra were buffer corrected.

### Bis-ANS-assay

Changes in the surface hydrophobicity were monitored by the binding of 4,4’-dianilino-1,1’-binaphthyl-5,5’-disulfonic acid (bis-ANS, Molecular Probes) resulting in a fluorescent signal upon excitation at 370 nm. Trx2 was diluted to a final concentration of 3 μM in 10 mM KH_2_PO_4_, pH 7.0 containing 15 μM bis-ANS. The emission spectrum was recorded from 400–600 nm at 25°C and 42°C using a Hitachi F4500 fluorescence spectrophotometer. All spectra were buffer corrected.

### Gel filtration chromatography

Recombinant Trx2 and 5S-Trx2 (450 μM to 900 μM) in 50 mM sodium phosphate, 300 mM NaCl, pH 8.0 were preincubated for 30 min at 25°C in the presence or absence of 25 mM DTT. After centrifugation for 45 min at 13,000 rpm and 4°C, 25 to 50 μl of the clear protein solution was loaded onto a Superdex 75 10/300 GL column equilibrated in 50 mM sodium phosphate, 150 mM sodium chloride, pH 7.0 ± 1 mM DTT and connected to an ÄKTA Purifier system (GE Healthcare). Gel filtration was performed at room temperature at a velocity of 0.3 ml/min and detection at 280, 320, and 420 nm. Ribonuclease A (13.7 kDa), chymotrypsinogen A (25 kDa +/- 25%), ovalbumin (44 kDa), conalbumin (75 kDa), and alcohol dehydrogenase (150 kDa) served as molecular mass standards. To measure the chaperone activity of the different oligomeric species, a 200 μM solution of Trx2_red_, Trx2_ox_ or 5S-Trx2 was loaded onto a Superdex 200 10/300 GL column (GE Healthcare) equilibrated with 40 mM HEPES, 140 mM NaCl, pH 7.5 ± 5 mM DTT. The gel filtration was run at a flow-rate of 0.5 ml/min at 4°C using an Äkta-FPLC system. Absorption at 280 nm was detected and the individual elution fractions were studied by SDS-PAGE and in the luciferase aggregation assay.

### Size-exclusion chromatography with multi-angle light scattering (SEC-MALS)

SEC-MALS was performed to determine the absolute molecular mass of 5S-Trx2. Typically 100 μl of 80 μM recombinant 5S-Trx2 was injected into a Superdex 200 10/300 column connected to an ÄKTA Purifier. This system was coupled to a light scattering detector (DAWN8+ HELEOS, Wyatt Technology) and a refractometer (Optilab tREX, Wyatt Technology) to measure the absolute refractive index of the solution. Runs were carried out at 4°C and a flow rate of 0.4 ml/min. Data were analyzed using the manufacturer supplied software (ASTRA 6.1, Wyatt Technology). The Rayleigh ratio and differential refractive index were plotted against the elution volume.

### Insulin reduction assay

The assay was conducted essentially as described previously [26]. In a total volume of 200 μl of 100 mM potassium phosphate, 2 mM EDTA, pH 7.0, various concentrations of recombinant Trx2, 5S-Trx2 and/or Tpx as positive control were incubated with 2–3 mM DTT for 20 min at room temperature. The reaction was started by adding 600 μl of a 1 mg/ml insulin solution in buffer, resulting in a final concentration of 130 μM insulin. The increase in turbidity was monitored at 650 nm and 37°C. To generate Trx2 or Tpx species in which the cysteines were blocked, the pre-reduced proteins were incubated with 15 mM N-ethylmaleimide (NEM) for 30 min at room temperature.

### Luciferase and citrate synthase aggregation assays

The effect of Trx2_red_, Trx2_ox_ and 5S-Trx2 on the aggregation of unfolding proteins was studied using the (1) luciferase and (2) citrate synthase aggregation assays. (1) To measure thermal unfolding, a fresh solution of 12 μM luciferase (Promega) was prepared in 40 mM MOPS, 50 mM KCl, pH 7.5 (assay buffer) and diluted to a final concentration of 0.1 μM in assay buffer pre-heated to 44°C to initiate protein aggregation under continuous stirring. (2) 12 μM Citrate synthase (Sigma-Aldrich) was denatured by overnight incubation at room temperature in 40 mM HEPES, pH 7.5 containing 6 M guanidine hydrochloride. To follow protein aggregation, the denatured citrate synthase was diluted to a final concentration of 0.075 μM in 40 mM HEPES, pH 7.5 at 30°C under continuous stirring. Light scattering was monitored (λ_ex/em_ = 360 nm) in a Hitachi F4500 fluorescence spectrophotometer equipped with a temperature-controlled cuvette holder and stirrer. The maximum in light scattering signal was reached after (1) 15 min and (2) 4 min of incubation and was set to 100%. To study the effect of Trx2 on the protein aggregation, different molar ratios of Trx2 were added into the assay buffer.

### Chaperone-dependent reactivation of thermally unfolded luciferase

A freshly prepared solution of 12 μM luciferase in 40 mM MOPS, 50 mM KCl, pH 7.5 (assay buffer) was diluted to 0.1 μM in assay buffer and incubated for 20 min at 42°C either alone or in the presence of 2 μM Trx2_red_, Trx2_ox_ or 5S-Trx2. As a positive control, luciferase was incubated in the presence of 2 μM DnaK, 0.4 μM DnaJ and 2 μM GrpE. For refolding, the reaction was cooled down to 25°C for 10 min and supplemented with 2 mM MgATP and 0.1 mg/ml BSA. Since none of the Trx2 variants mediated reactivation of luciferase on their own, 2 μM DnaK, 0.4 μM DnaJ, and 2 μM GrpE were added to the refolding reaction. At defined time points, 5 μl aliquots were taken to measure luciferase activity in a 96-well plate. Luminescence was measured upon adding 95 μl of 100 mM KH_2_PO_4_, 25 mM glycyl glycine, 200 μM EDTA, pH 7.5, containing 2 mM MgATP, 0.5 mg/ml BSA and 70 μM luciferin at 25°C using the FLUOstar Omega microplate reader (BMG Labteck).

## Supporting information

S1 Table Primers used in this study. (DOCX)

S1 Fig Comparison of *T. brucei* Trx2 with trypanosomatid orthologs and classical thioredoxins. (**A**) Multiple sequence alignment comparing *T*. *brucei* trx2 with other thioredoxins using the CLUSTAL O (1.2.4) program with minor manual adjustments. (**B**) Percentage of identical (similar) residues obtained by pairwise alignment of the sequences with the EMBOSS Needle program. (**C**) Neighbor-joining tree of Trx-like sequences from Kinetoplastid and non-Kinetoplastid organisms without distance corrections, and (**D**) rooted phylogenetic tree for Trx-like proteins from Kinetoplastids.
(DOCX)

S2 Fig Cloning of different BS and PC Trx2 cKO cell lines. BS and PC cKO *T*. *brucei* cell lines that ectopically expressed different C-terminally myc_2_-tagged versions of Trx2 were generated as outlined in Materials and methods. (**A**) The scheme shows the binding sites of the primers (and expected amplicon sizes) to confirm (a) absence of the endogenous alleles and correct insertion of the (b) blasticidin (bla) resistance cassette and (c) puromycin (pac) resistance cassette. (**B**) BS and PC parasites were transfected with constructs to replace the two *trx2* alleles by puromycin (pac) and blasticidin (bla) resistance genes. Genomic DNA from double-resistant cell lines as well as WT BS *T*. *brucei* was subjected to PCR analysis to verify the absence or presence of the endogenous alleles using Trx2 IN-F and Trx2-R as primers (a in **A**). The gel electrophoresis revealed that all cell lines had retained a *trx2* copy. Subsequently, cKO *T*. *brucei* cell lines that ectopically expressed (**C**) WT-Trx2 or (**D**) Trx2 species in which either the two putative active site (C63/66S) or all five cysteines (5S) were replaced by serine residues were generated. (**C**) PCR analysis with Trx2 IN-F and Trx2-R as primers (a in **A**) confirmed the loss of the endogenous *trx2* alleles in BS and PC WT-Trx2 cKO cell lines. Genomic DNA from WT parasites or a single-KO (sKO) cell line served as positive controls. (**D**) Genomic DNA from BS and PC C63/66S-Trx2 cKO and 5S-Trx2 cKO cell lines as well as WT BS *T*. *brucei* was subjected to PCR analysis with the three primer combinations depicted in (**A**). The DNA fragments were separated on 1% agarose gels.
(TIF)

S3 Fig Analysis of C63/66S-Trx2 cKO cell lines. (**A**) BS and (**B**) PC cKO cells expressing C63/66S-Trx2 were cultured in the absence or presence of 1 μg/ml Tet, alongside WT parasites. Every 24 h, the cells were counted and diluted back to the starting density of 1 x 10^5^ (BS) or 5 x 10^5^ (PC) cells/ml. The left graphs show the percentage of cells at each time point relative to the start density set as 100%. A paired t-test was used to evaluate significant differences between the C63/66S-Trx2 cKO cells grown in the presence or absence of Tet at each time point (* = p < 0.05). The right graphs provide the corresponding cumulative densities. The values are the mean ± SD from each three independent BS and PC cell lines. (**C**) Western blot analyses of total lysates from 1 x 10^7^ cells from three PC C63/66S-Trx2 cKO cell lines. Five days after tet withdrawal, the protein was still detectable indicating that ectopic expression of the mutant Trx2 was not tightly regulated. Due to the specificity of the myc-antibodies we assume that all bands detected represent differently processed forms of the protein. Aldolase served as loading control. (**D**) Immunofluorescence microscopy of induced PC C63/66S-Trx2 cKO cells using anti-myc antibodies (green). The mitochondrion was stained with MitoTrackerRed (red) and the kinetoplast and nucleus with DAPI (blue). Merge, overlay of the three signals. Phase, phase contrast image.
(TIF)

S4 Fig Parasitemia of mice infected with Trx2 RNAi and cKO *T. brucei* cell lines. Groups of five animals fed with (filled symbols) or without (open symbols) oxytetracycline in the drinking water were infected with 10^4^ WT parasites, a Tet-inducible Trx2 RNAi cell line (RNAi) or Tet-inducible cKO cell lines expressing either WT-Trx2 (WT-cKO) or 5S-Trx2 (5S-cKO). The blood parasitemia was monitored at intervals over the course of the experiment and is shown for different days for each individual animal together with the median value for the respective group (horizontal line). Asterisks denote statistically significant differences (*p* < 0.05, Mann Whitney test) with the corresponding *p* values above the line.
(TIF)

S5 Fig Effect of redox stressors in PC WT parasites and Trx2 mutants. (**A**) WT parasites were incubated with 4 mM DTT for 0, 1 or 3 h and total lysates of different amounts of cells subjected to Western blot analysis with Trx2 antibodies and re-probed for aldolase as loading control. The band above the aldolase band probably represents the residual of the intense band cross-reacting with the Trx2 antiserum. The level of Trx2 was unaffected by the DTT treatment. (**B**) WT parasites and 5S-Trx2 cKO cells kept in the presence of Tet were diluted to 5 x 10^5^ cells/ml and incubated in the absence (control) or presence of 150 μM DTT. After different times, viable cells were counted. The percentage of cells relative to the starting density (mean ± SD) from three independent cell lines is depicted. (**C**) WT parasites and 5S-Trx2 cKO cell lines maintained in the presence of Tet were diluted to 5 x 10^5^ cells/ml and treated with H_2_O_2_. After different times, living cells were counted. If necessary, the cultures were diluted back to the starting density and the stressor concentration was restored. In the left graph, cells were treated with high concentrations of H_2_O_2_ and short-term cell viability was monitored. The percentage of cells relative to the starting density set 100% is depicted. In the right graph, the cumulative cell densities upon long-term cultivation of the cells in the presence of H_2_O_2_ are depicted. All data for the 5S-Trx2 cKO cells are the mean ± SD obtained with three independent cell lines. The analysis of the WT parasites was done several times with very similar outcomes. The experiments were repeated a second time yielding identical results. (**D**) WT-Trx2 cKO and 5S-Trx2 cKO were pre-cultured for five days in Tet-free medium. Under these conditions, the ectopically expressed proteins became depleted and the 5S-Trx2 cKO cells displayed a significant proliferation defect (see main text, Fig 3B and 3D). WT-Trx2 cKO ± Tet, 5S-Trx2 cKO ± Tet as well as WT parasites were treated with paraquat, and after different times living cells were counted. The graph depicts the cumulative cell densities obtained. The experiment was repeated using two other clones of WT-Trx2 cKO and 5-S-Trx2 cKO cells. All data showed that paraquat impaired the proliferation of WT parasites, WT-Trx2 cKO cells and 5S-Trx2 cKO cells to the same degree. Also towards 5S-Trx2 cKO -Tet cells which displayed a proliferation defect, paraquat had only an additive effect. After 72 h (in total 8 days after Tet-removal, see also Fig 3D), the cells restarted to proliferate indicating that the regulation of the Tet-inducible expression system was lost, even in the presence of the stressor.
(TIF)

S6 Fig Generation and analysis of recombinant *T. brucei* WT-, C63/66S- and 5S-Trx2 species. SDS-PAGE followed by Coomassie staining of **(A)** different purification steps of recombinant WT-Trx2, (**B**) purified full length (fl) Trx2 and **(C)** WT-Trx2, C63/66S-Trx2 and 5S-Trx2 with or without DTT in sample buffer.
(TIF)

S7 Fig Heat-induced chaperone activity of Trx2_red_ and 5S-Trx2 mitigates aggregation of chemically denatured citrate synthase. (**A**) Aggregation of chemically denatured citrate synthase (75 nM) at 30.5°C was followed by measuring light scattering at 360 nm in the absence or presence of different molar ratios of Trx2_red_, Trx2_ox_ or 5S-Trx2, pre-incubated for 5 min at either 25°C (upper panel) or 40°C (lower panel). (**B**) Aggregation of citrate synthase in the absence of Trx2 was set as 100% and the percentage of light scattering after 240 min in the presence of a 20:1 or 40:1 molar ratio of Trx2 to luciferase was calculated from at least three independent assays.
(TIF)

S8 Fig Trx2 is unable to refold heat-inactivated luciferase. (**A**) Luciferase (0.1 μM) was heat-inactivated for 20 min at 42°C in the absence or presence of a 20:1 molar ratio of Trx2_red_, Trx2_ox_ and 5S-Trx2 treated accordingly. After cooling the samples to 25°C for 10 min, 0.1 mg/ml BSA and 2 mM MgATP were added (grey arrow). At indicated time points, aliquots were collected, and luciferase activity was measured. The luciferase activity measured before the inactivation at 42°C was set to 100%.
(TIF)

S9 Fig Temperature-dependent changes in the secondary structure of Trx2. The far-UV circular dichroism spectra of Trx2_red_, Trx2_ox_ and 5S-Trx2 were recorded at 25°C and 42°C. Temperature-induced changes were followed at 222 nm while increasing the temperature by 1°C/min. All spectra were buffer corrected.
(TIF)

S10 Fig Co-immunoprecipitation of different Trx2 species. Lysates from 1 x 10^9^ PC cKO cells expressing C-terminally myc-tagged versions of WT-Trx2, C63/66S-Trx2 or 5S-Trx2 alongside WT parasites as background control were loaded onto anti-myc agarose beads as described in S1 Text. Aliquots of the total cell lysate, unbound fraction, third wash and elution fraction were subjected to Western blot analyses with anti-myc antibodies. The elution fractions displayed similar levels for the three Trx2-myc_2_ species in accordance with their comparable enrichment in the quantitative mass analysis.
(TIF)

S1 Text Searching for interaction partners of Trx2. (DOCX)
