# Supplementary material for: An essential thioredoxin-type protein of Trypanosoma brucei acts as redox-regulated mitochondrial chaperone
Source: PLoS Pathog. 2019 Sep 26;15(9):e1008065. doi: 10.1371/journal.ppat.1008065 (PMC6783113; doi:10.1371/journal.ppat.1008065)
Supplement: S1 Table — (DOCX) [file ppat.1008065.s001.docx]

S1 Table

| **Primer name** | **Sequence** | **Purpose** |
| --- | --- | --- |
| Trx2 Long *Nco*I-F | *ccatgg*GTTCTGCTACCGTGGGCCTCC | Cloning of full length *trx2* into pET vector |
| Trx2 Short *NcoI*-F | *ccatgg*GTAGCGGTGCCTGCTTGAGCG | Cloning of *trx2* lacking the first 32 amino acids into pET vector |
| Trx2 *Acc*65I-R | *ggtacc*TCAATTTGTCGCCCCACCCTC | Cloning of both *trx2* versions into pET vector |
| Trx2i-*Hpa*I | atc*gttaac*TAAGCCGTGCATATCCATC | Amplification of *trx2* fragments for cloning into the pHD678 vector for RNAi |
| Trx2i-*EcoR*I_1 | ata*gaattc*gtgggaacgctgcgtatatc |  |
| Trx2i-*Hind*III | atc*aagctt*TAAGCCGTGCATATCCATC |  |
| Trx2i-*EcoR*I_2 | ata*gaattc*CCTTCCAAGTGACCAATAATG |  |
| Trx2-5’UTR-*Xho*I | attc*ctcgag*CTCCGAATTGTAATTGTGACG | Amplification of *trx2* 5’UTR |
| Trx2-5’UTR-*Hind*III | tcg*aagctt*GTATTAAAACTCCTCACGGG |  |
| Trx2-3’UTR-*Pst*I | taa*ctgcag*TTATCGCAGGCAGACGCGTG | Amplification of *trx2* 3’UTR |
| Trx2-3’UTR-*Not*I | at*gcggccgc*CCACTAAGCACTCAGCGATTG |  |
| Trx2-5’OUT-F | CCTTTTGCCCAACGCGCATG | To confirm insertion of the blasticidin or puromycin resistance cassettes into the *trx2* locus in cKO cells |
| Puro-R | *cggctaagc*TCAGGCACCGGGCTTGCGGG |  |
| Bla-R | AGGGCAGCAATTCACGAATC |  |
| Trx2 IN-F | GCTTGTGCCACCCGTGAGG | To verify deletion of the *trx2* alleles by PCR |
| Trx2-R | TCAATTTGTCGCCCCACCC |  |
| Trx2-myc_6_-*Hind*III-F | *aagctt*ATGTCTGCTACCGTGGGC | Cloning of *trx2* with C-terminal myc_6_ tag into the pRPa vector |
| Trx2-myc_6_-*Xba*I-R | *tctaga*ATTTGTCGCCCCACCCTC |  |
| Trx2-myc_2_-*Hind*III-F | taa*aagctt*ATGTCTGCTACCGTGGGC | Cloning of *trx2* with C-terminal myc_2_ tag into the pHD1700 vector |
| Trx2-myc_2_-*BamH*I-R | taa*ggatcc*ATTTGTCGCCCCACCCTC |  |
| Trx2-C36S-F | ggagcggtgccagcttgagcggg | Mutation of Cys 36 to Ser |
| Trx2-C36S-R | Cccgctcaagctggcaccgctcc |  |
| Trx2-C36S-pET-F | ggtagcggtgccagcttgagcgggc | Mutation of Cys 36 to Ser in the pET-MBP-Trx2 vector |
| Trx2-C36S-pET-R | gcccgctcaagctggcaccgctacc |  |
| Trx2-C63+66S-F | CACTAATCGGAGTAAGCCGAGCATATCCATC | Mutation of Cys 63 and 66 to Ser |
| Trx2-C63S+66-R | GATGGATATGCTCGGCTTACTCCGATTAGTG |  |
| Trx2-C92S-F | cgtttcctcttccattccccaatctagctcatcacacgcttattc | Mutation of Cys 92 to Ser |
| Trx2-C92S-R | gaataagcgtgtgatgagctagattggggaatggaagaggaaacg |  |
| Trx2-C169S-F | catttgtggcatatcgcagtggtcgcattattggtc | Mutation of Cys 169 to Ser |
| Trx2-C169S-R | gaccaataatgcgaccactgcgatatgccacaaatg |  |

Overhang bases and restriction sites are given in lower case and both lower case and italics, respectively. Serine codons replacing cysteine codons are underlined. Unless otherwise stated, primers used to mutate cysteine to serine residues were used in both pHD1700-Trx2 and pET-MBP-Trx2 vectors. F and R, forward and reverse primer, respectively.
