# Supplementary material for: An essential thioredoxin-type protein of Trypanosoma brucei acts as redox-regulated mitochondrial chaperone
Source: PLoS Pathog. 2019 Sep 26;15(9):e1008065. doi: 10.1371/journal.ppat.1008065 (PMC6783113; doi:10.1371/journal.ppat.1008065)
Supplement: S1 Fig — (A) Multiple sequence alignment comparing T. brucei trx2 with other thioredoxins using the CLUSTAL O (1.2.4) program with minor manual adjustments. (B) Percentage of identical (similar) residues obtained by pairwise alignment of the sequences with the EMBOSS Needle program. (C) Neighbor-joining tree of Trx-like sequences from Kinetoplastid and non-Kinetoplastid organisms without distance corrections, and (D) rooted phylogenetic tree for Trx-like proteins from Kinetoplastids. (DOCX) [file ppat.1008065.s002.docx]

**A**

*T. brucei* Trx2(427) -----------------*MSATVGLRRYATSILNVSRLRQEAIRGYALRR*SGACLSGQLRI 43

*T. brucei* Trx2(927) -----------------MSATVGLRRYVTSILNVSRLRQEAIRGYALRRSGACLSGQLRI 43

*T. evansi* Trx2 -----------------MSATVGLRRYATSILNVSRLRQEAIRGYALRRSGACLSGQLRI 43

*T. cruzi* Trx2 -------MKVAVGFLQHCLVRRRTGTSVTTVFSTAPAASLSSWTCATRRHIATFRGHSRE 53

*L. major* Trx2 172--SPNDSAAFADTAGQKQRSPVADRTCDSSPLPPSGTEPLAATPPAPVYSFHGNPQE 226

*L. braz*iliensis Trx2 173--SPNGGAASTGTARQNLGSSAATPTCDSSSAPLSSTETLVARPPARVFSFRGNPHE 227

*T. brucei* Trx1 -------------------------------------------------MSVVDVYSVEQ 11

*T. cruzi* Trx1 -------------------------------------------------MPVVDVYSVEQ 11

Human Trx1 ---------------------------------------------------VKQIESKTA 9

*E. coli* Trx1 ------------------------------------------------SDKIIHLTDDSF 12

*T. brucei* Trx2(427) FTDH--VNADAAVLVYFHTNRCKPCISITRALEEMETEKEQGVVSS------------SI 89

*T. brucei* Trx2(927) FTDH--VNADAAVLVYFHTNRCKPCISITRALEEMETEKEQGVVSS------------SI 89

*T. evansi* Trx2 FTDH--VNADAAVLVYFHTNRCKPCISITRALEEMETEKEQGVVSS------------SI 89

*T. cruzi* Trx2 FLDE--VNREKVALVYFYAPWREPRNRVYVKLEEFLNKGRPAVRGGSEATSSTSLASSSL 111

*L. major* Trx2 LQDR--L-RGARALVFFYKASCAPCAVIRSKLLHAVAGAGGG-GGSATVAPPTSTEPATE 282

*L. braz*iliensis Trx2 LQDC--L-RGAKVLVFFYKTSCAPCTAIRSKLLHALAGAGVAGDGGATATPPTSTVFATE 284

*T. brucei* Trx1 FRNI--MSEDILTVAWFTAVWCGPCKTIERPME--------------------------- 42

*T. cruzi* Trx1 FREI--VNEDLLTVAWFTAVWCGPCKTIERAME--------------------------- 42

Human Trx1 FQEALDAAGDKLVVVDFSATWCGPCKMIKPFFH--------------------------- 42

*E. coli* Trx DTDV--LKADGAILVDFWAEWCGPCKMIAPILD--------------------------- 43

: :. * * : :

*T. brucei* Trx2(427) PQCSSSHAYSQEDTLGLTQRLLR---------LL-G---ITFADVAV------------- 123

*T. brucei* Trx2(927) PQCSSSHACSQEDTLGLTQRLLR---------LL-G---ITFADVAV------------- 123

*T. evansi* Trx2 PQCSSSHAYSQEDTLGLTQRLLR---------LL-G---ITFADVAV------------- 123

*T. cruzi* Trx2 SSPSAAAASPSKDPAA--EGTLQ---------PG-A---LADPKSRV------------- 143

*L. major* Trx2 VAGIDGVPSPLLDHLQKNQRQPDSPTSVSAAAPMAAYAEAKDAKAARATTASLPATVMTL 342

*L. braz*iliensis Trx2 DSGIDGSSPPLSAPLQLYQNNRERPTRGLTTAPVAADTEATDAAAVV-----ATTATMTV 339

*L. infantum* Trx2 MTL **3**

*T. brucei* Trx1 ------------------------------------------------------------ 42

*T. cruzi* Trx1 ------------------------------------------------------------ 42

Human Trx1 ------------------------------------------------------------ 42

*E.coli* Trx1 ------------------------------------------------------------ 43

*T. brucei* Trx2(427) -SPE-------ALGVLREGVTRGVRIISVDTDENPVISALHDIRSVPTFVAYRCGRIIGH 175

*T. brucei* Trx2(927) -SPE-------ALGVLREGVTRGVRIISVDTDENPVISALHDIRSVPTFVAYRCGRIIGH 175

*T. evansi* Trx2 -SPE-------ALGVLREGVTRGVRMISVDTDENPVISALHDIRSVPTFVAYRCGRIIGH 175

*T. cruzi* Trx2 -DPEVLLKSVREFCVRARANGRTVRIIPVNTDENPKLGALHDIRSVPTFVTYRDGRIVGR 202

*L. major* Trx2 EDQKSCDVACRFLGNATKPFAHSVILITVNTNANAEVTALHDIRSLPTFMAYRNGCIVGR 402

*L. braziliensis* Trx2 EEQKACNVACRFLGGVAKPFPHSVILLMVDTNANAEVTALHDIRSLPTFMAYRNGCIIGR 399

*L. infantum* Trx2 EDQKSCDVACRFLGDATKSFAHSVILITVDTNANAEVTALHDIRSLPTFMAYRNGCIVGR 63

*T. brucei* Trx1 --------------KIAYEFPT-VKFAKVDADNNSEIVSKCRVLQLPTFIIARSGKMLGH 87

*T. cruzi* Trx1 --------------KITFEFPT-VRFAKVDADNNSEIVSKCKVLQLPTFMLVKNGKLIGY 87

Human Trx1 --------------SLSEKYSN-VIFLEVDVDDCQDVASECEVKCMPTFQFFKKGQKVGE 87

*E.coli* Trx1 --------------EIADEYQGKLTVAKLNIDQNPGTAPKYGIRGIPTLLLFKNGEVAAT 89

: . :: : : :**: : * .

*T. brucei* Trx2(427) LEGASEQKLQELVSKLLVEEGGATN------------------------------ 200

*T. brucei* Trx2(927) LEGASEQKLQELVSKLLVEEGGATN------------------------------ 200

*T. evansi* Trx2 LEGASEQKLQELVSKLLVEEGGATN------------------------------ 200

*T. cruzi* Trx2 VEGFSEDQIEQLVKELLQEDSGDTPVTDQAKRNWKSKECEETKK---GEKK---- 250

*L. major* Trx2 FEGSHEDEINKLVDLLAENSTKGSPATDNGTPSPPPQQQGATSKRVSGD------ 451

*L. braziliensis* Trx2 FEGSHEDEIDKLVDLLTQDSTERSPTTDNEKASQQQQQQGTTSSPVSGDYDSHHP 454

*L. infantum* FEGSQEDEINKLVDLLAENSTERSPTTDNRTPSPLPQQEGATSIRVSGD 112

*T. brucei* Trx1 VIGANPGMLRQKLRDIIKDN----------------------------------- 107

*T. cruzi* Trx1 VIGANLDTLKRKIREIISNT----------------------------------- 107

Human Trx1 FSGANKEKLEATINELV-------------------------------------- 104

*E.coli* Trx1 KVGALSK---GQLKEFLDANLA--------------------------------- 108

* : :





**
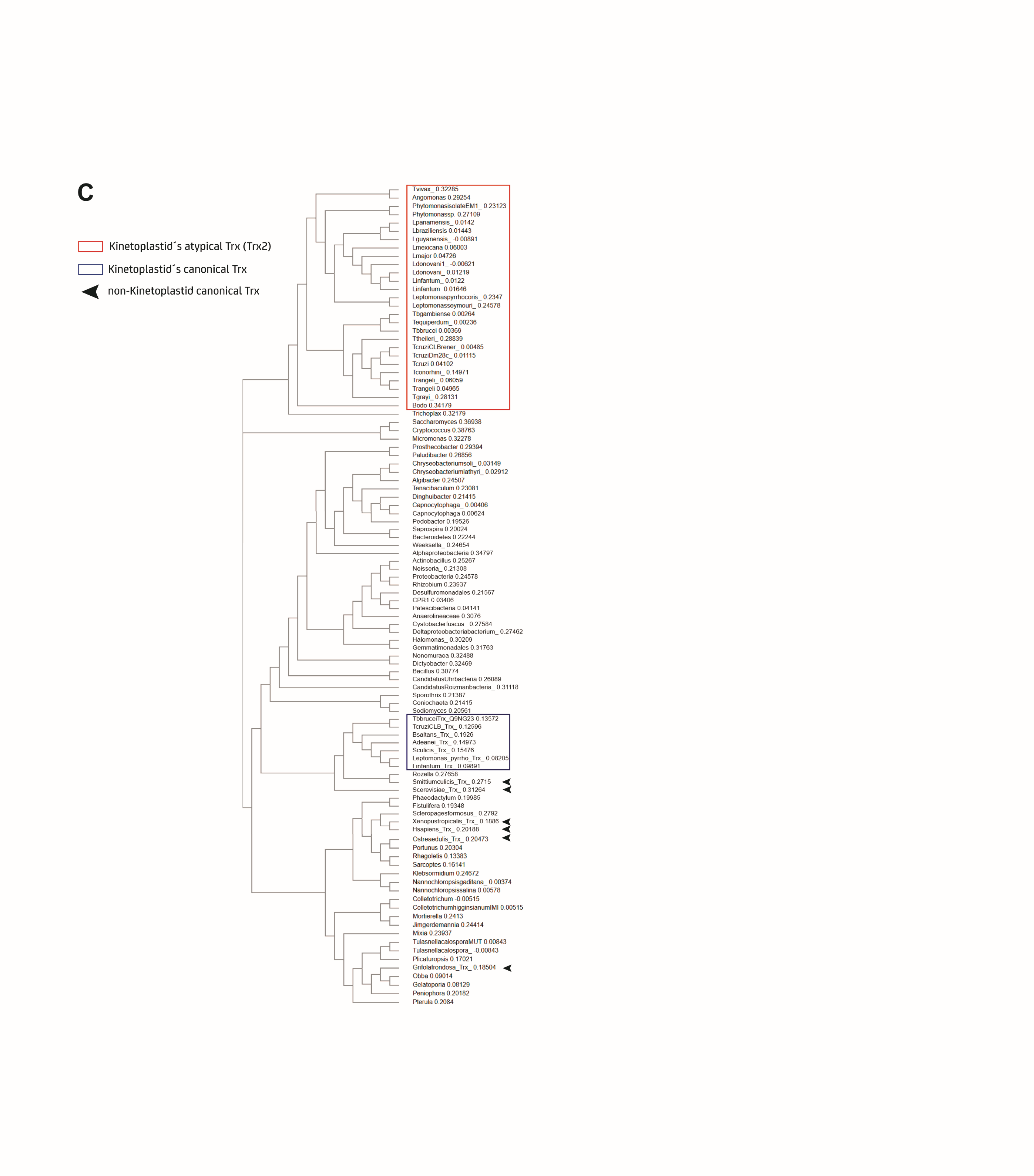
**


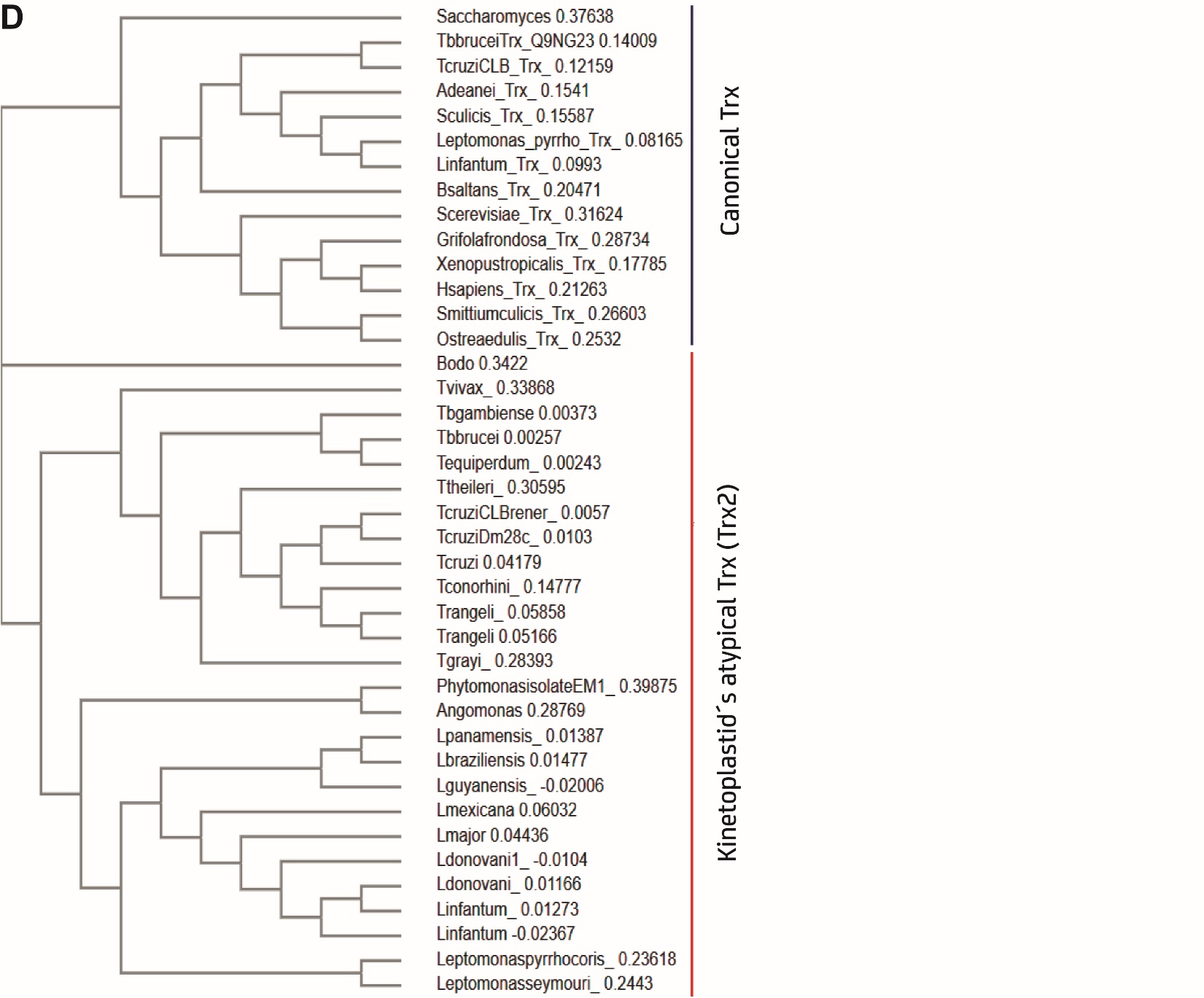


**S1 Fig. Comparison of *T. brucei* Trx2 with trypanosomatid orthologs and classical thioredoxins.** (**A**) The proteins were compared using the CLUSTAL O (1.2.4) multiple sequence alignment program with minor manual adjustments. The putative mitochondrial pre-sequence that is absent in the recombinant *T. brucei* Trx2 is given in italics and underlined. Cysteine residues are highlighted by a yellow background. The methionine that in the sequence from *Leishmania infantum* and some other *Leishmania* species has been annotated as start residue is underlined (for details see main text). *T. brucei* Trx2 (427), Tb427.03.4240; *T. brucei* Trx2 (927), Tb927.3.4240; *T. evansi* Trx2, TevSTIB805.3.4480; *T. cruzi* Trx2, TcCLB.509617.60; *L. major* Trx2*, Leishmania major*, LmjF.29.1650; *L. braziliensis* Trx2, LbrM.29.1760; *L. infantum* Trx2, LinJ29.1790; *T. brucei* Trx1, Tb927.9.3370; *T. cruzi* Trx1, TcCLB.511577.83; Human Trx1, AAF86466; *E. coli* Trx1, *Escherichia coli* Trx1, CDU40139. (**B**) The percentage of identical (similar) residues as obtained by pairwise alignment of the sequences with the EMBOSS Needle program. With the aim to identify protein homologues of atypical and canonical Trx, a Blast-P search against all non-redundant GenBank CDS translations was performed using *T. brucei* Trx2 (Tb427.03.4240) and *T. brucei* Trx1 (Q9NG23), respectively, as query sequences. A subset of sequences from each group and representative of different organisms was subjected to phylogenetic analysis: (**C**) Neighbor-joining tree of Trx-like sequences from Kinetoplastid and non-Kinetoplastid organisms without distance corrections, and (**D**) Rooted phylogenetic tree for Trx-like proteins from Kinetoplastids. In both cases, the branch length value is indicated next to the species name. The phylogenetic analysis was performed using the Clustal Omega tool (Sievers et al. 2011).
