# Supplementary figures and images for: An essential thioredoxin-type protein of Trypanosoma brucei acts as redox-regulated mitochondrial chaperone
Source: PLoS Pathog. 2019 Sep 26;15(9):e1008065. doi: 10.1371/journal.ppat.1008065 (PMC6783113; doi:10.1371/journal.ppat.1008065)

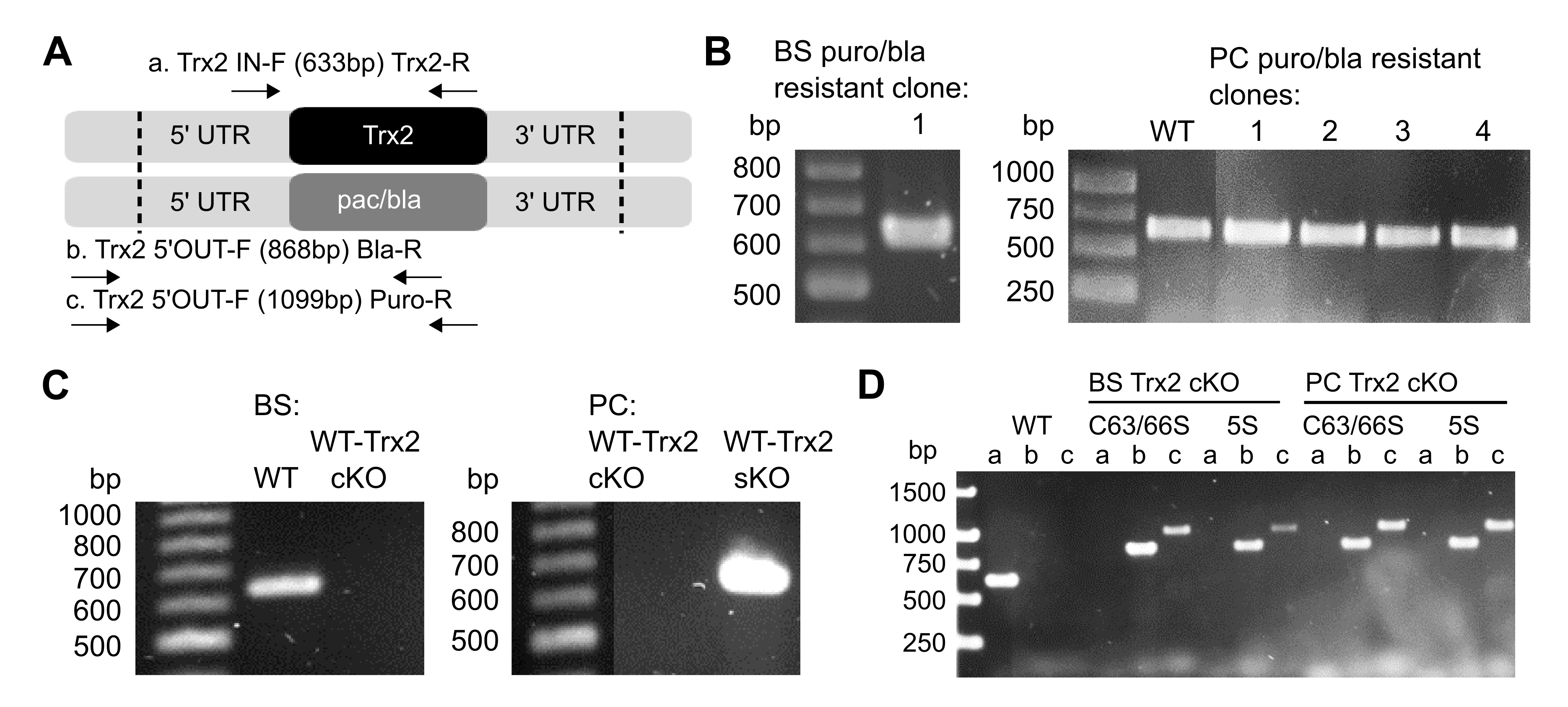

Supplement: S2 Fig — BS and PC cKO T. brucei cell lines that ectopically expressed different C-terminally myc2-tagged versions of Trx2 were generated as outlined in Materials and methods. (A) The scheme shows the binding sites of the primers (and expected amplicon sizes) to confirm (a) absence of the endogenous alleles and correct insertion of the (b) blasticidin (bla) resistance cassette and (c) puromycin (pac) resistance cassette. (B) BS and PC parasites were transfected with constructs to replace the two trx2 alleles by puromycin (pac) and blasticidin (bla) resistance genes. Genomic DNA from double-resistant cell lines as well as WT BS T. brucei was subjected to PCR analysis to verify the absence or presence of the endogenous alleles using Trx2 IN-F and Trx2-R as primers (a in A). The gel electrophoresis revealed that all cell lines had retained a trx2 copy. Subsequently, cKO T. brucei cell lines that ectopically expressed (C) WT-Trx2 or (D) Trx2 species in which either the two putative active site (C63/66S) or all five cysteines (5S) were replaced by serine residues were generated. (C) PCR analysis with Trx2 IN-F and Trx2-R as primers (a in A) confirmed the loss of the endogenous trx2 alleles in BS and PC WT-Trx2 cKO cell lines. Genomic DNA from WT parasites or a single-KO (sKO) cell line served as positive controls. (D) Genomic DNA from BS and PC C63/66S-Trx2 cKO and 5S-Trx2 cKO cell lines as well as WT BS T. brucei was subjected to PCR analysis with the three primer combinations depicted in (A). The DNA fragments were separated on 1% agarose gels. (TIF) [file ppat.1008065.s003.tif]

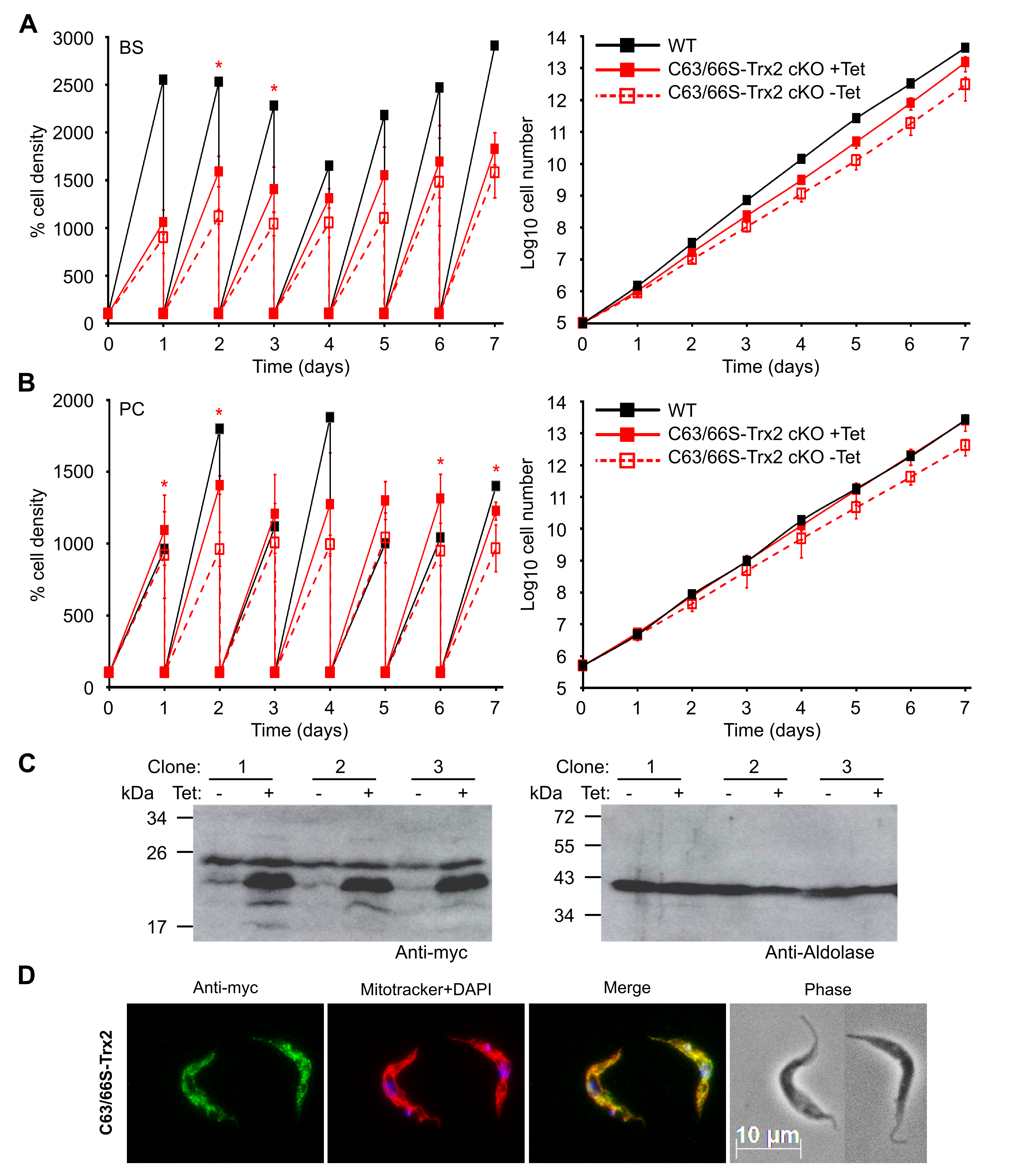

Supplement: S3 Fig — (A) BS and (B) PC cKO cells expressing C63/66S-Trx2 were cultured in the absence or presence of 1 μg/ml Tet, alongside WT parasites. Every 24 h, the cells were counted and diluted back to the starting density of 1 x 10^5^ (BS) or 5 x 10^5^ (PC) cells/ml. The left graphs show the percentage of cells at each time point relative to the start density set as 100%. A paired t-test was used to evaluate significant differences between the C63/66S-Trx2 cKO cells grown in the presence or absence of Tet at each time point (* = p < 0.05). The right graphs provide the corresponding cumulative densities. The values are the mean ± SD from each three independent BS and PC cell lines. (C) Western blot analyses of total lysates from 1 x 10^7^ cells from three PC C63/66S-Trx2 cKO cell lines. Five days after tet withdrawal, the protein was still detectable indicating that ectopic expression of the mutant Trx2 was not tightly regulated. Due to the specificity of the myc-antibodies we assume that all bands detected represent differently processed forms of the protein. Aldolase served as loading control. (D) Immunofluorescence microscopy of induced PC C63/66S-Trx2 cKO cells using anti-myc antibodies (green). The mitochondrion was stained with MitoTrackerRed (red) and the kinetoplast and nucleus with DAPI (blue). Merge, overlay of the three signals. Phase, phase contrast image. (TIF) [file ppat.1008065.s004.tif]

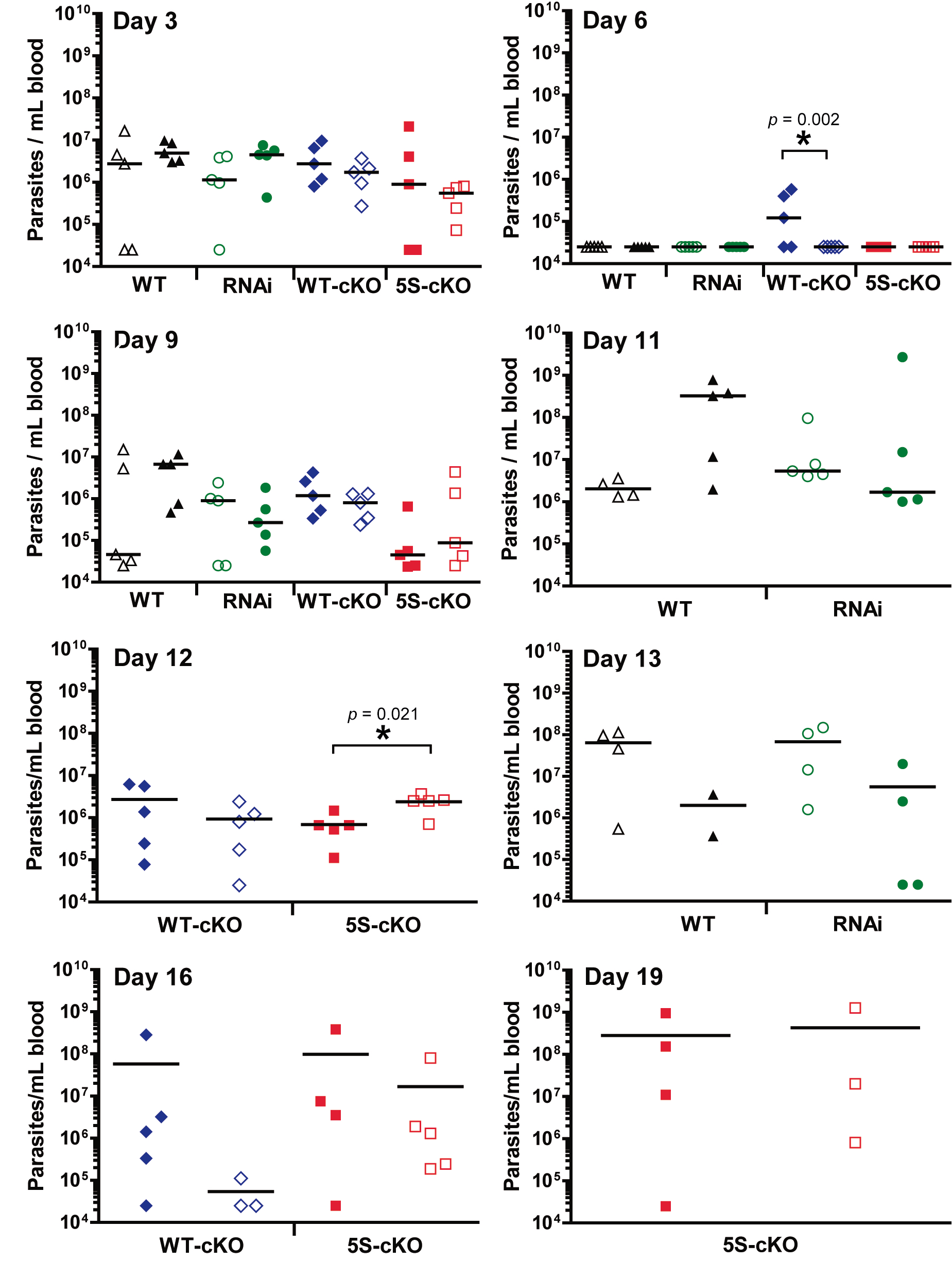

Supplement: S4 Fig — Groups of five animals fed with (filled symbols) or without (open symbols) oxytetracycline in the drinking water were infected with 104 WT parasites, a Tet-inducible Trx2 RNAi cell line (RNAi) or Tet-inducible cKO cell lines expressing either WT-Trx2 (WT-cKO) or 5S-Trx2 (5S-cKO). The blood parasitemia was monitored at intervals over the course of the experiment and is shown for different days for each individual animal together with the median value for the respective group (horizontal line). Asterisks denote statistically significant differences (p < 0.05, Mann Whitney test) with the corresponding p values above the line. (TIF) [file ppat.1008065.s005.tif]

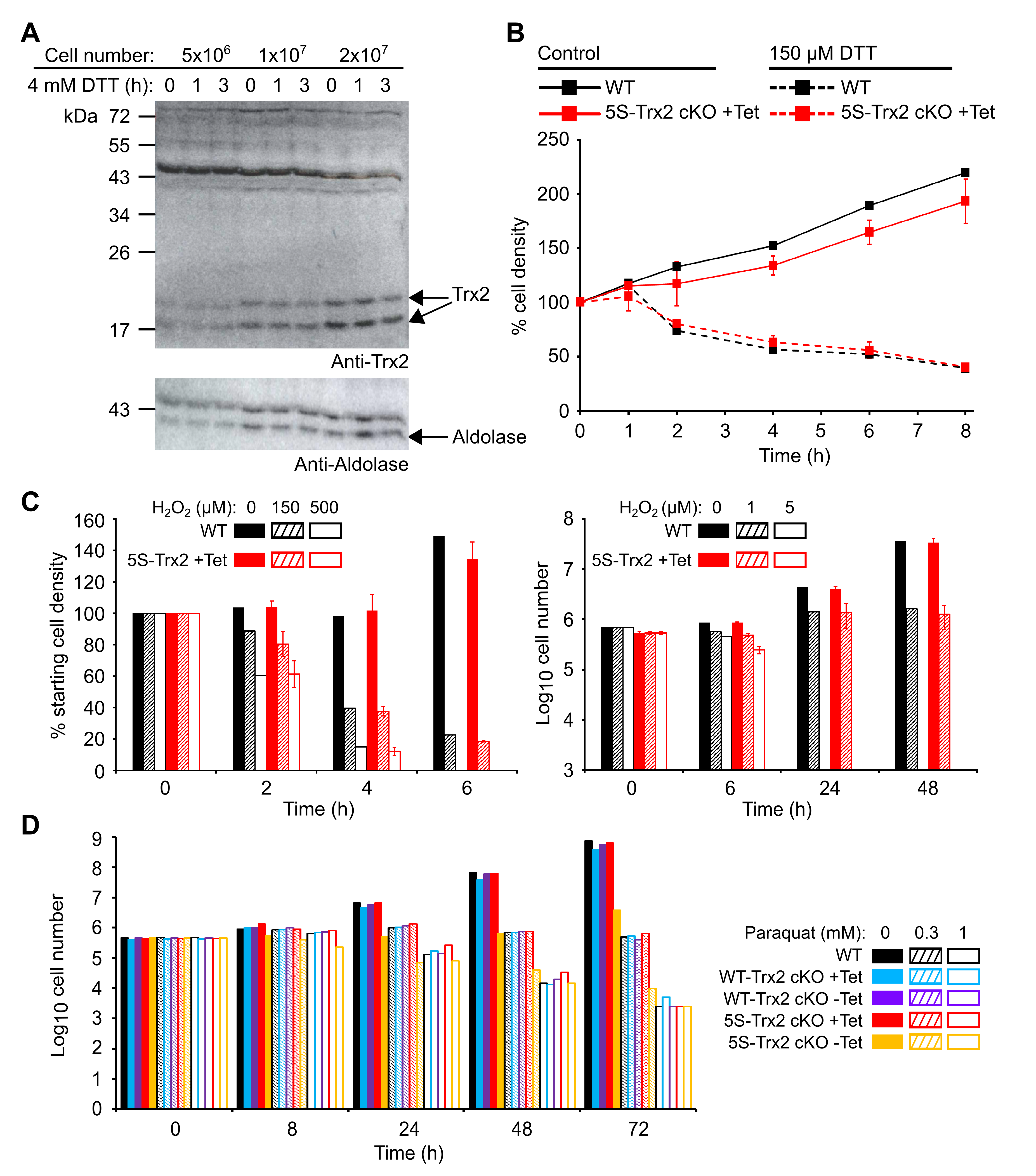

Supplement: S5 Fig — (A) WT parasites were incubated with 4 mM DTT for 0, 1 or 3 h and total lysates of different amounts of cells subjected to Western blot analysis with Trx2 antibodies and re-probed for aldolase as loading control. The band above the aldolase band probably represents the residual of the intense band cross-reacting with the Trx2 antiserum. The level of Trx2 was unaffected by the DTT treatment. (B) WT parasites and 5S-Trx2 cKO cells kept in the presence of Tet were diluted to 5 x 10^5^ cells/ml and incubated in the absence (control) or presence of 150 μM DTT. After different times, viable cells were counted. The percentage of cells relative to the starting density (mean ± SD) from three independent cell lines is depicted. (C) WT parasites and 5S-Trx2 cKO cell lines maintained in the presence of Tet were diluted to 5 x 10^5^ cells/ml and treated with H2O2. After different times, living cells were counted. If necessary, the cultures were diluted back to the starting density and the stressor concentration was restored. In the left graph, cells were treated with high concentrations of H2O2 and short-term cell viability was monitored. The percentage of cells relative to the starting density set 100% is depicted. In the right graph, the cumulative cell densities upon long-term cultivation of the cells in the presence of H2O2 are depicted. All data for the 5S-Trx2 cKO cells are the mean ± SD obtained with three independent cell lines. The analysis of the WT parasites was done several times with very similar outcomes. The experiments were repeated a second time yielding identical results. (D) WT-Trx2 cKO and 5S-Trx2 cKO were pre-cultured for five days in Tet-free medium. Under these conditions, the ectopically expressed proteins became depleted and the 5S-Trx2 cKO cells displayed a significant proliferation defect (see main text, Fig 3B and 3D). WT-Trx2 cKO ± Tet, 5S-Trx2 cKO ± Tet as well as WT parasites were treated with paraquat, and after different times living cells were count [file ppat.1008065.s006.tif]

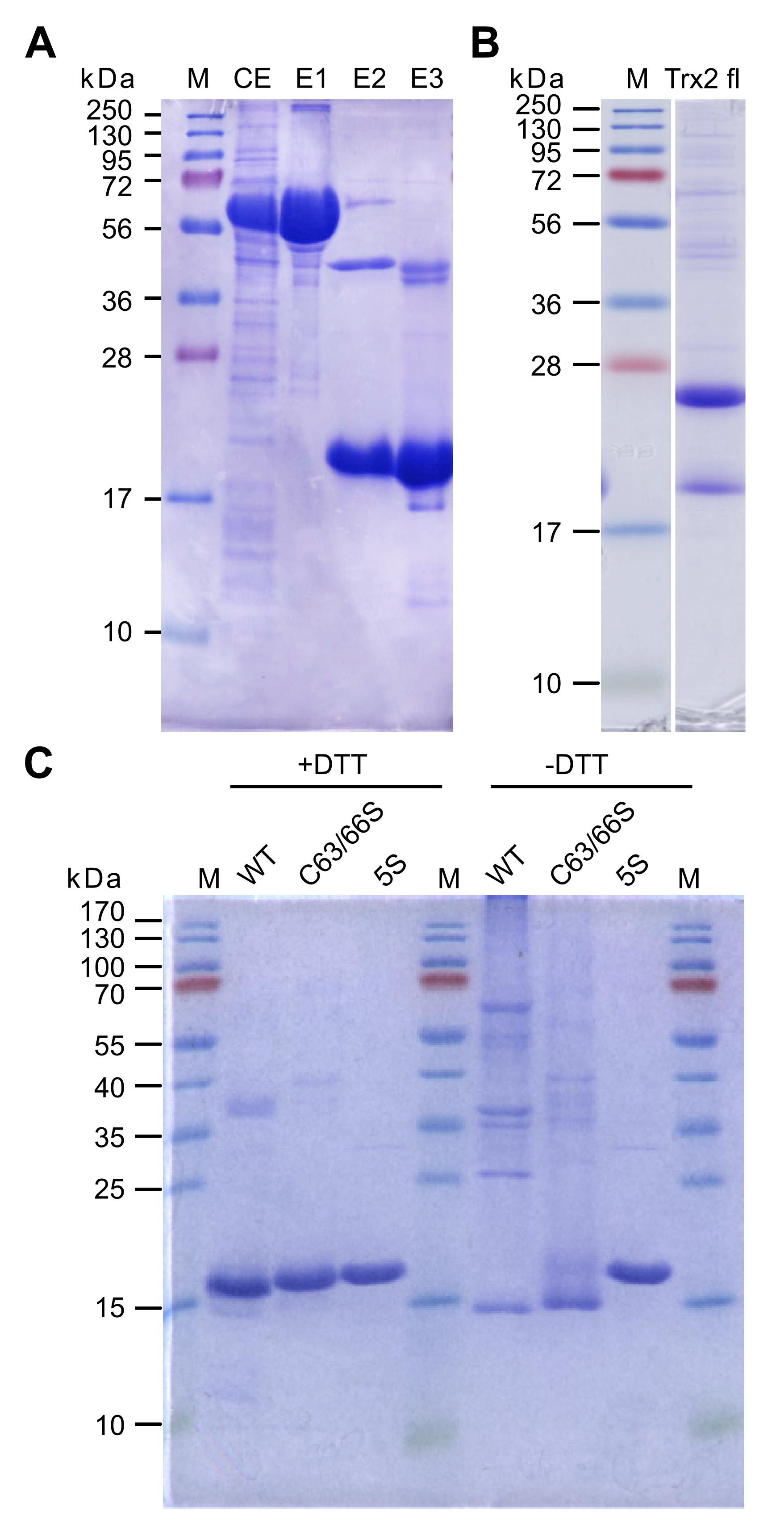

Supplement: S6 Fig — SDS-PAGE followed by Coomassie staining of (A) different purification steps of recombinant WT-Trx2, (B) purified full length (fl) Trx2 and (C) WT-Trx2, C63/66S-Trx2 and 5S-Trx2 with or without DTT in sample buffer. (TIF) [file ppat.1008065.s007.tif]

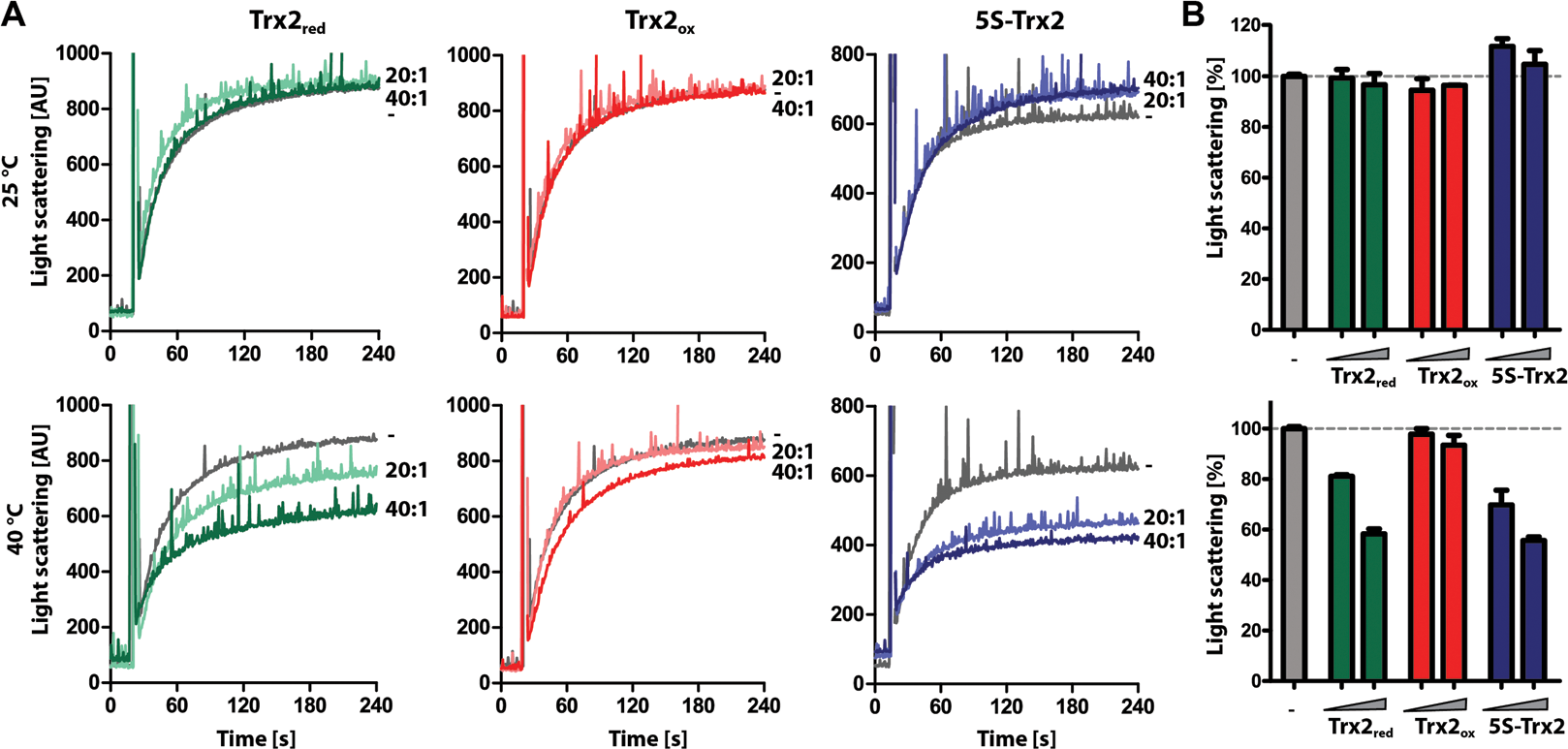

Supplement: S7 Fig — (A) Aggregation of chemically denatured citrate synthase (75 nM) at 30.5°C was followed by measuring light scattering at 360 nm in the absence or presence of different molar ratios of Trx2red, Trx2ox or 5S-Trx2, pre-incubated for 5 min at either 25°C (upper panel) or 40°C (lower panel). (B) Aggregation of citrate synthase in the absence of Trx2 was set as 100% and the percentage of light scattering after 240 min in the presence of a 20:1 or 40:1 molar ratio of Trx2 to luciferase was calculated from at least three independent assays. (TIF) [file ppat.1008065.s008.tif]

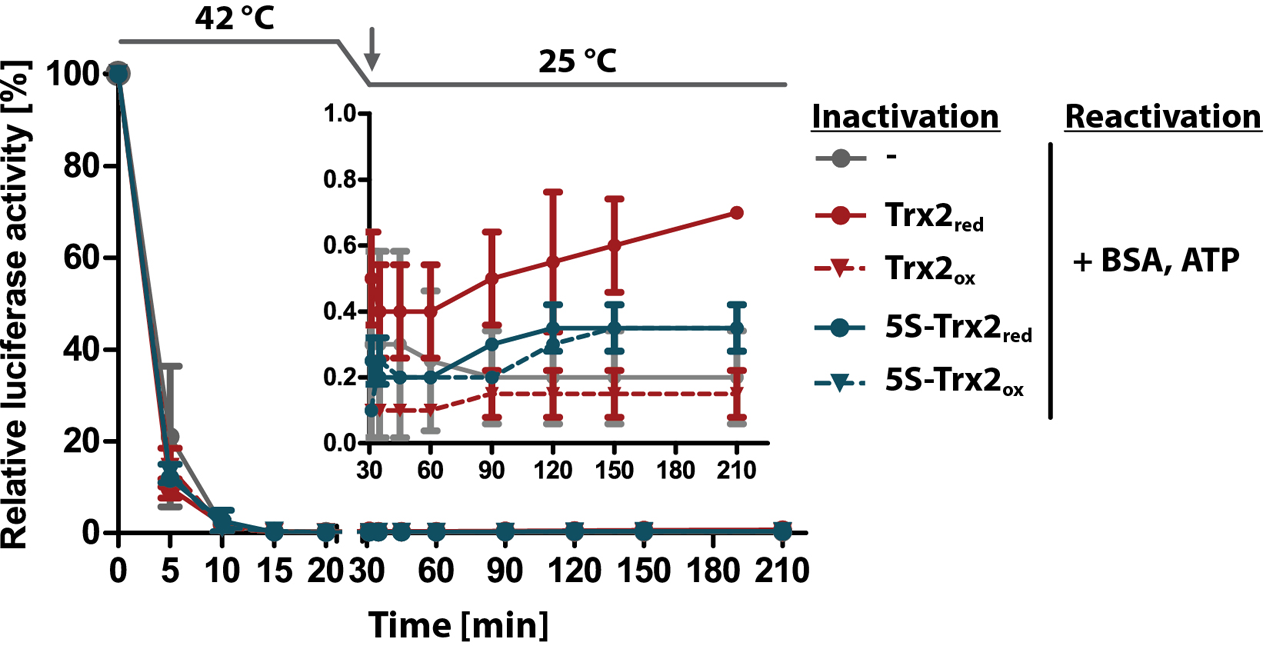

Supplement: S8 Fig — (A) Luciferase (0.1 μM) was heat-inactivated for 20 min at 42°C in the absence or presence of a 20:1 molar ratio of Trx2red, Trx2ox and 5S-Trx2 treated accordingly. After cooling the samples to 25°C for 10 min, 0.1 mg/ml BSA and 2 mM MgATP were added (grey arrow). At indicated time points, aliquots were collected, and luciferase activity was measured. The luciferase activity measured before the inactivation at 42°C was set to 100%. (TIF) [file ppat.1008065.s009.tif]

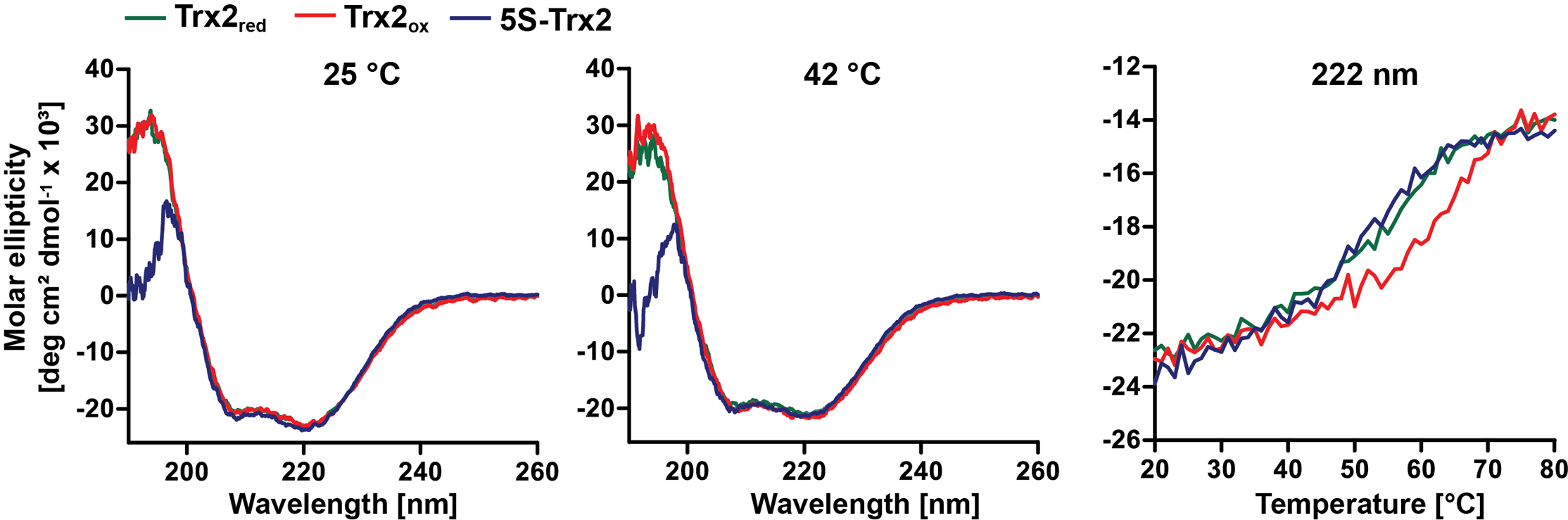

Supplement: S9 Fig — The far-UV circular dichroism spectra of Trx2red, Trx2ox and 5S-Trx2 were recorded at 25°C and 42°C. Temperature-induced changes were followed at 222 nm while increasing the temperature by 1°C/min. All spectra were buffer corrected. (TIF) [file ppat.1008065.s010.tif]

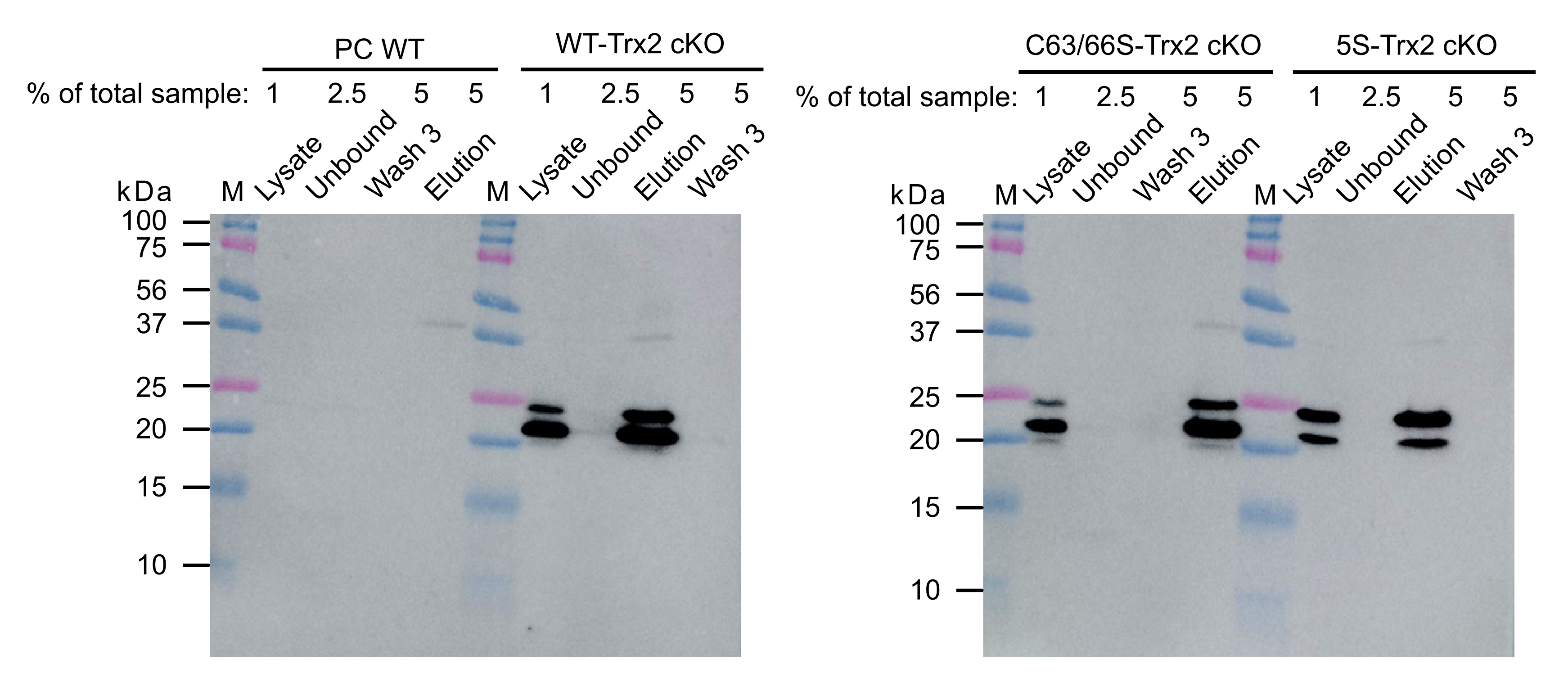

Supplement: S10 Fig — Lysates from 1 x 10^9^ PC cKO cells expressing C-terminally myc-tagged versions of WT-Trx2, C63/66S-Trx2 or 5S-Trx2 alongside WT parasites as background control were loaded onto anti-myc agarose beads as described in S1 Text. Aliquots of the total cell lysate, unbound fraction, third wash and elution fraction were subjected to Western blot analyses with anti-myc antibodies. The elution fractions displayed similar levels for the three Trx2-myc2 species in accordance with their comparable enrichment in the quantitative mass analysis. (TIF) [file ppat.1008065.s011.tif]
