# Supplementary material for: An essential thioredoxin-type protein of Trypanosoma brucei acts as redox-regulated mitochondrial chaperone
Source: PLoS Pathog. 2019 Sep 26;15(9):e1008065. doi: 10.1371/journal.ppat.1008065 (PMC6783113; doi:10.1371/journal.ppat.1008065)
Supplement: S1 Text — (DOCX) [file ppat.1008065.s012.docx]

**S1 Text, Searching for interaction partners of Trx2**

**Methods**

1 x 10^9^ of C-terminally myc_2_-tagged WT-Trx2, C63/66S-Trx2 and 5S-Trx2 cKO cell lines as well as PC WT cells were centrifuged, washed with PBS and snap frozen. All subsequent steps were conducted at 4°C. Cell pellets were reconstituted in immunoprecipitation (IP) lysis buffer (50 mM Tris, pH 8.0, 120 mM NaCl, 0.5% NP-40, 2 mM DTT and 50 µg/ml leupeptin) and lysed by passing through a 25 gauge needle 20 times. The lysate was centrifuged at 13,000 rpm for 10 min and the supernatant was incubated with 200 µl anti-c-myc-coupled agarose beads (Biomol) with rotation for 4 h. After 3-5 washes with IP wash buffer (50 mM Tris, pH 8.0, 120 mM NaCl and 0.5% NP-40), proteins were eluted from the beads by 30 min rotation in 0.1 M glycine, pH 2.5. To validate the procedure, aliquots of the cell lysate, unbound fraction, washes and elution fractions were analyzed by Western blotting using anti-myc antibodies. The eluates were concentrated, and the buffer was exchanged to 50 mM ammonium bicarbonate, pH 7.5, using a 3,000 Da Amicon filter (Merck). Subsequently, the elution fractions from the WT-Trx2, C63/66S-Trx2 and 5S-Trx2 cKO cells as well as the WT cell lysates were subjected to SDS-PAGE on a short 15% gel, run for 15 min and stained with Coomassie Blue. The gel lanes were cut into three fractions and the proteins in-gel digested with trypsin as described previously (Chi et al. 2011). The eluted peptides were desalted using ZipTip-μC18 material (Merck Millipore) and re-suspended in 0.1% (v/v) formic acid. The peptide solutions from the three gel fractions of the same sample were combined and the peptides of the four Trx2 CoIP samples subjected to nLC-MS/MS analysis using an Orbitrap Fusion (Thermo Fisher Scientific) coupled to a TriVersa NanoMate (Advion, Ltd.) as described previously (Kublik et al. 2016). In total, 5 μl peptide solution was separated at a constant flow of 300 nl/min on a 15 cm analytical column (Acclaim PepMap 100 RSLC, 2 µm C18 particles, nanoViper connections, 75 µm x 25 cm Thermo Scientific) at 35°C on a Dionex Ultimate 3000 nano-LC system (Dionex/Thermo Fisher Scientific) using 0.1% formic acid (solvent A) and 80% acetonitrile, 0.08% formic acid (solvent B) and a linear 90 min gradient of 4% to 55% solvent B. Peptide identification was conducted by Proteome Discoverer (version 2.2, Thermo Fisher Scientific) using the SequestHT search engine as described (Seidel et al. 2018) and the annotated protein databases for the *T. brucei* 427 and 927 strains accessed from TriTrypDB (current release, last modified 24.08.18). Peptides were considered to be identified with high and medium confidence at a target false discovery rate (FDR) of ≤0.01 and ≤0.05, respectively, based on the q-values. Identified proteins were quantified by the 'Percursor Ions Quantifier' implemented in Proteome Discoverer 2.2 as based on peak intensities to estimate the abundance of the Trx2 CoIP proteins in the analysed CoIP samples. For determination of fold-enrichment of Trx2 interaction partners, the total abundance of each protein detected in the CoIP eluates of the three myc-tagged cell lines was divided by the abundance detected in the WT cell background. Proteins which were enriched in at least two of the three myc-tagged cell lines were considered. Proteins were cross-referenced against a list of non-specific proteins identified by BioID with Myc-BirA*-CIF1 as the bait (Zhou et al. 2018).

**Results**

WT-Trx2, C63/66S-Trx2 or 5S-Trx2 cKO cells, alongside WT cells, were subjected to co-immunoprecipitation. The myc-tagged proteins were detected in the total lysate and elution fractions derived from the three cKO cell lines but not in the WT control cells, validating the overall procedure (S10 Fig). The eluates were subjected to label-free quantitative mass spectrometry*.* Trx2 species were enriched between 40 and 112-fold. In addition, 264 (320) proteins were found to be enriched >3-fold and 61 (95) proteins were enriched >5-fold, when searching against the *T. brucei* 427 (or 927) database. To reduce the number of putatively non-specifically binding proteins, we repeated the analysis but included two further washing steps of the agarose beads until we could no longer detect any protein in the wash fraction. Again, the myc-tagged Trx2 proteins were enriched between 100 and 183-fold. For *T. brucei* 427 (or 927), we identified 292 (283) and 144 (138) proteins with >3 and >5-fold enrichment, respectively. Clearly, the more stringent washing did not reduce the number of proteins. To further evaluate the data obtained, we compared the proteins which were enriched >5-fold in both co-immunoprecipitation experiments. Proteasome regulatory ATPase subunit 2 (Tb427tmp.02.1220, Tb927.11.3740) was the only protein which was identified as being ≥5-fold enriched in both co-immunoprecipitation experiments by searching the *T. brucei* 427 and 927 databases. We did not find any strong evidence from the current literature or from the TrypTag database (<http://tryptag.org>) for a mitochondrial localisation. Therefore, we conclude that our approach did not yield a specific interaction partner of Trx2.

**References**

Chi BK, Gronau K, Mader U, Hessling B, Becher D, Antelmann H. S-bacillithiolation protects against hypochlorite stress in Bacillus subtilis as revealed by transcriptomics and redox proteomics. Mol Cell Proteomics. 2011;10(11):M111 009506. doi: 10.1074/mcp.M111.009506.

Kublik A, Deobald D, Hartwig S, Schiffmann CL, Andrades A, von Bergen M, et al. Identification of a multi-protein reductive dehalogenase complex in Dehalococcoides mccartyi strain CBDB1 suggests a protein-dependent respiratory electron transport chain obviating quinone involvement. Environmental Microbiology. 2016;18(9):3044-56. doi: 10.1111/1462-2920.13200.

Seidel K, Kühnert J, Adrian L. The Complexome of Dehalococcoides mccartyi Reveals Its Organohalide Respiration-Complex Is Modular. Frontiers in Microbiology. 2018;9:1130-. doi: 10.3389/fmicb.2018.01130.

Sievers F., Wilm A., Dineen D., Gibson T.J., Karplus K., Li W., Lopez R., McWilliam H., Remmert M., Söding J., Thompson J.D. and Higgins D.G. (2011) Fast, scalable generation of high-quality protein multiple sequence alignments using Clustal Omega. Mol. Syst. Biol. 7:539. doi: 10.1186/1471-2105-6-29.

Zhou Q, An T, Pham KTM, Hu H, Li Z. The CIF1 protein is a master orchestrator of trypanosome cytokinesis that recruits several cytokinesis regulators to the cytokinesis initiation site. Journal of Biological Chemistry. 2018;293(42):16177-92. doi: 10.1074/jbc.RA118.004888.
